# Engagement of sialylated glycans with Siglec receptors on suppressive myeloid cells inhibits anticancer immunity via CCL2

**DOI:** 10.1038/s41423-024-01142-0

**Published:** 2024-03-06

**Authors:** Ronja Wieboldt, Michael Sandholzer, Emanuele Carlini, Chia-wei Lin, Anastasiya Börsch, Andreas Zingg, Didier Lardinois, Petra Herzig, Leyla Don, Alfred Zippelius, Heinz Läubli, Natalia Rodrigues Mantuano

**Affiliations:** 1 Laboratory for Cancer Immunotherapy, Department of Biomedicine, University Hospital and University of Basel, Basel, Switzerland; 2 Functional Genomics Center Zurich, ETH Zurich, Zurich, Switzerland; 3 Bioinformatics Core Facility, Department of Biomedicine, University of Basel and Swiss Institute of Bioinformatics, Basel, Switzerland; 4 Department of Thoracic Surgery, University Hospital Basel, Basel, Switzerland; 5 Laboratory of Cancer Immunology, Department of Biomedicine, University Hospital and University of Basel, Basel, Switzerland; 6 Division of Oncology, University Hospital Basel, Basel, Switzerland

**Keywords:** sialoglycans, Sialic acid-binding immunoglobulin-like lectin, tumor microenvironment, myeloid derived suppressor cells, Tumour immunology, Tumour immunology

## Abstract

The overexpression of sialic acids on glycans, called hypersialylation, is a common alteration found in cancer cells. Sialylated glycans can enhance immune evasion by interacting with sialic acid-binding immunoglobulin-like lectin (Siglec) receptors on tumor-infiltrating immune cells. Here, we investigated the effect of sialylated glycans and their interaction with Siglec receptors on myeloid-derived suppressor cells (MDSCs). We found that MDSCs derived from the blood of lung cancer patients and tumor-bearing mice strongly express inhibitory Siglec receptors and are highly sialylated. In murine cancer models of emergency myelopoiesis, Siglec-E knockout in myeloid cells resulted in prolonged survival and increased tumor infiltration of activated T cells. Targeting suppressive myeloid cells by blocking Siglec receptors or desialylation strongly reduced their suppressive potential. We further identified CCL2 as a mediator involved in T-cell suppression upon interaction between sialoglycans and Siglec receptors on MDSCs. Our results demonstrated that sialylated glycans inhibit anticancer immunity by modulating CCL2 expression.

## Introduction

Immunotherapy has revolutionized the field of cancer treatment, but only a subset of patients responds to this therapy and many relapse over time. Primary or acquired therapeutic resistance can be facilitated by the suppressive tumor microenvironment (TME), which helps cancer cells evade immunosurveillance [1]. The overexpression of sialic acids on glycans in cancer cells can lead to hypersialylation. Hypersialylation can drive cancer progression by mediating the stability of cell surface receptors on cancer cells and integrin-mediated interactions and enhancing immune evasion by engaging sialic acid-binding immunoglobulin-like lectin (Siglec) receptors on immune cells, modulating antigen presentation, interacting with selectin receptors and modulating the stability of surface proteins on immune cells [2–4]. Siglec receptors are highly expressed by different immune cells. The majority of Siglec receptors are part of the CD33-related inhibitory Siglec family, which mainly transduces signals via cytosolic immunoreceptor tyrosine-based inhibitory motifs (ITIMs) [5]. Recent studies have highlighted the importance of inhibitory Siglec receptors for the regulation of various immune cells, including T cells, natural killer (NK) cells, dendritic cells (DCs) and macrophages [6–10]. The Siglec-sialoglycan axis in cancer is a potential immune checkpoint target for cancer immunotherapy that can be modulated by blocking Siglec receptors or cleaving sialoglycans via sialidases [11]. For example, a sialidase linked to a tumor-directed antibody was successfully used in vivo and improved tumor control by repolarizing macrophages in the TME [12, 13].

Pathologically activated myeloid cells are known to promote an immunosuppressive TME and represent a promising target for cancer therapy [14]. This closely related family of myeloid suppressors mainly consists of tumor-associated macrophages (TAMs) and myeloid-derived suppressor cells (MDSCs). Induced by aberrant myelopoiesis, MDSCs are a heterogeneous group of immature myeloid cells that are involved in the suppression of various immune cells via the secretion of suppressive cytokines or direct cell‒cell interactions [15]. MDSCs can be subdivided into monocytic MDSCs (mMDSCs) and granulocytic or polymorphonuclear MDSCs (gMDSCs or PMN-MDSCs), which are identified according to their monocytic or granulocytic myeloid cell lineage markers [16]. In addition to Siglec-3/CD33 being a phenotypic marker expressed by all MDSCs, recent studies have further described the expression of Siglec-5, Siglec-7, and Siglec-9 in human glioma patient MDSCs and that of Siglec-E on murine MDSCs [12, 17]. Nevertheless, little is known about the effect of sialoglycan ligands and Siglec receptors on MDSCs and their expression in healthy and diseased conditions. Here, we investigated the role of the Siglec-sialoglycan axis in MDSC generation and function in the TME of humans and mice and their expression patterns in lung tumor patients and healthy individuals.

## Results

### Myeloid cells express inhibitory CD33-related Siglec receptors in cancer

Although no exclusive MDSC markers are known, MDSCs can be identified by the coexpression of phenotypic surface markers that vary between mice and humans. All murine MDSCs express Gr1 and CD11b and can be subdivided into Ly6G^+^Ly6C^low^ gMDSCs and Ly6C^high^ mMDSCs [18]. In humans, gMDSCs are phenotypically characterized as CD33^+^CD11b^+^HLA-DR^low/-^CD15^+^CD14^-^ and mMDSCs as CD33^+^CD11b^+^HLA-DR^low/-^CD15^-^CD14^+^ [16]. All human MDSCs express Siglec-3/CD33 as a marker [16], but little is known about the expression of additional Siglec receptors on MDSCs in human cancer patients and their functional relevance [17].

**Fig. 1:**
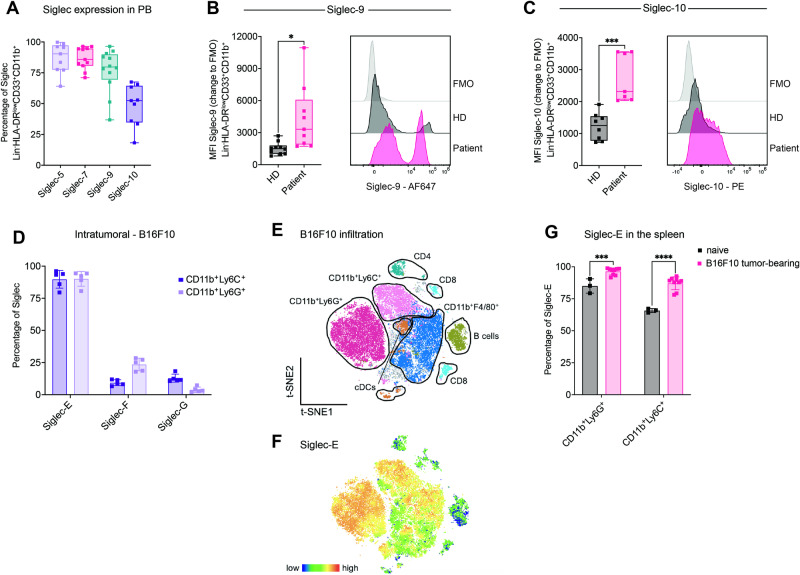
Myeloid cells express Siglec receptors in humans and mice. **A** Percentages of Lin^-^HLA-DR^low^CD33^+^CD11b^+^ cells expressing Siglec-5, Siglec-7, Siglec-9 and Siglec-10 in the peripheral blood (PB) from lung cancer patients detected by flow cytometry. *n* = *9–12 donors per group*. **B** MFI of Siglec-9 and (**C**) Siglec-10 in CD45^+^Lin^-^HLA-DR^low^CD33^+^CD11b^+^ cells derived from healthy donor and lung cancer patient PB from (**A**). Exemplary results for each condition, including the fluorescence minus one (FMO) control, are shown on the right. The MFI is shown as the change in FMO and was determined by flow cytometry. *n* = *7–10 donors per group*. **D** Subcutaneously injected endpoint tumors from B16F10 melanoma engrafted mice were harvested and digested, and immune cell infiltration was assessed via multiparameter flow cytometry. Siglec-E, Siglec-F and Siglec-G expression was assessed on CD45^+^CD11b^+^Ly6G^+^ and CD45^+^CD11b^+^Ly6C cells. *n* = *5 mice*. **E** T-distributed stochastic neighbor embedding (t-SNE) projection of multicolor flow cytometry immunophenotyping of pooled infiltrating immune cells from B16F10 tumors. *n* = *5 mice*. **F** The Siglec-E expression intensity is shown as a color gradient from blue (low) to red (high). **G** Spleens from naïve and B16F10 melanoma tumor-bearing mice at the endpoint were collected and analyzed for Siglec-E expression via flow cytometry. *n* = *3–9 mice per group*. The data are presented as the mean ± SD. Two-tailed unpaired Student’s *t* test or multiple unpaired *t* tests (**G**) were used. *P < 0.05, **P < 0.01, ***P < 0.001, and ****P < 0.0001

Here, we investigated the expression of Siglec receptors on lung cancer patient-derived MDSCs as well as on myeloid cells from healthy donors (Fig. S1B) by gating on CD33^+^CD11b^+^HLA-DR^low/-^ cells (Fig. S1A–C). All of the patients were diagnosed with lung cancer and received no previous treatment (Table S2). Siglec-5, Siglec-7, Siglec-9, and Siglec-10 were highly expressed on MDSCs from lung cancer patients in the periphery and within the tumor (Figs. 1A, S1C). Siglec-9 and Siglec-10 expression was increased on patient MDSCs compared to myeloid cells from healthy donors in the peripheral blood (PB) (Fig. 1B, C). Siglec-5 and Siglec-7 was not differentially expressed on PB-derived MDSCs in cancer patients compared to healthy controls (Fig. S1D, E).

Next, we investigated whether our findings were similar in mice by analyzing mouse CD33-related inhibitory Siglec receptors on MDSCs, including Siglec-E, Siglec-F, and Siglec-G, which resemble potential functional paralogs of human Siglec-9, Siglec-8, and Siglec-10, respectively [19]. To this end, Siglec expression on tumor-infiltrating and spleen-derived MDSCs from tumor-bearing and naïve mice was assessed by analyzing the CD11b^+^Ly6G^+^ and CD11b^+^Ly6C^+^ populations, which, in the tumor context, are described as gMDSCs and mMDSCs, respectively [18] (Fig. S1F). High levels of Siglec-E were identified across infiltrating MDSCs in different tumor types, intermediate levels of Siglec-F were found only in gMDSCs, and Siglec-G was rarely expressed on both MDSC subtypes (Figs. 1D, S1G). The Siglec-E mean fluorescence intensity (MFI) was the highest for gMDSCs compared to all other tumor-infiltrating immune cells in B16F10 cells, followed by mMDSCs and TAMs (CD11b^+^F4/80^+^) (Fig. 1E, F). Furthermore, Siglec-E expression in the CD11b^+^Ly6G^+^ and CD11b^+^Ly6C^+^ populations in the spleens of B16F10- and EL4-bearing mice was increased compared to that in CD11b^+^Ly6G^+^ and CD11b^+^Ly6C^+^ populations in the spleens of naïve littermates (Figs. 1G, S1H). These results emphasize the increased expression of inhibitory Siglec receptors on suppressive myeloid cells of humans and mice in the context of cancer.

### Myeloid cells in cancer are hypersialylated

**Fig. 2:**
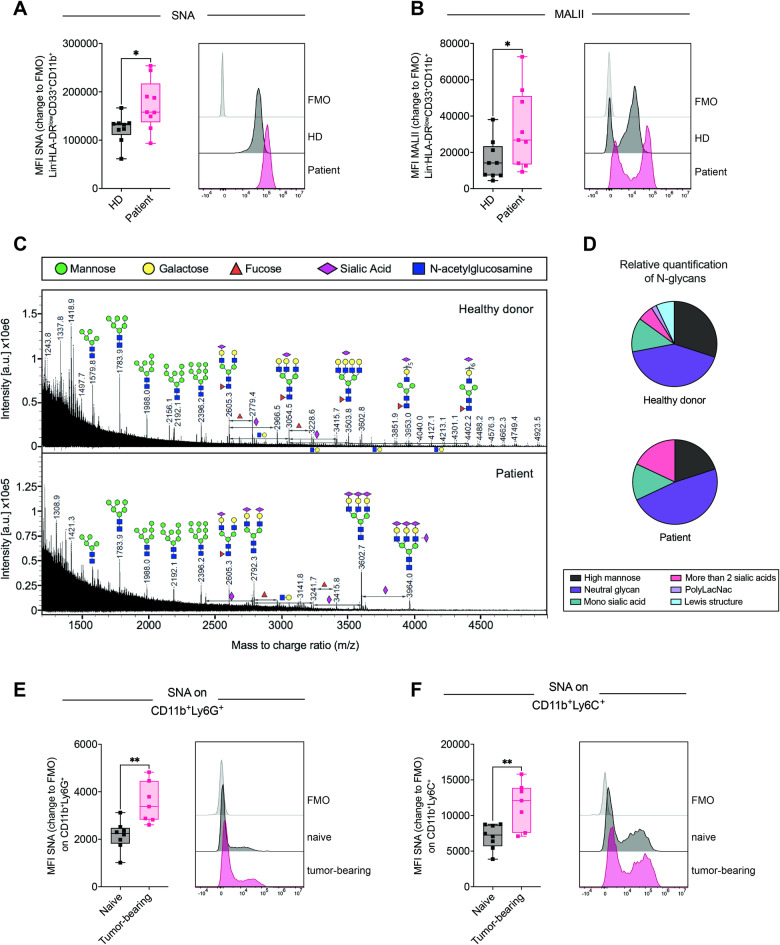
Myeloid cells in cancer are highly sialylated. **A** MFI of SNA or (**B**) MALII on PB-derived Lin^-^HLADR^low^CD33^+^CD11b^+^ cells from lung cancer patients and healthy controls. Exemplary results for each condition, including the fluorescence minus one (FMO) control, are shown on the right. The MFI is shown as the change in FMO and was determined by flow cytometry. *n* = *9 donors per group*. **C** MALDI-TOF mass spectra (m/z 1200–5000) of N-glycans isolated from fresh PB-derived CD33^+^ cells from healthy donors and lung cancer patients. The N-glycans were released by PNGaseF and permethylated prior to MALDI-TOF-TOF profiling. The main structures are depicted above the corresponding peaks. Assignments are based on the composition and knowledge of biosynthetic pathways. All molecular ions are [M + Na]^+^. The locations of residues above a bracket have not been clearly defined. **D** Relative quantification of N-glycan levels detected in cancer patient-derived and healthy donor-derived CD33^+^ cells from (**C**). *N* = *1*. **E** Fresh blood from B16F10 tumor-bearing mice and naïve wild-type mice was collected on Day 14 after tumor inoculation and analyzed for SNA gated on (**E**) CD45^+^CD11b^+^Ly6C^+^ or (**F**) CD45^+^CD11b^+^Ly6G^+^ cells. Representative results for each condition, including the FMO control, are shown on the right. The MFI is shown as the change in FMO. *7–8 mice per group*. The data are presented as the mean ± SD. Two-tailed unpaired Student’s *t* test was used. *P < 0.05, **P < 0.01, ***P < 0.001, and ****P < 0.0001

The binding of Siglec receptors to sialoglycan ligands has been described previously, and is involved in immune cell interactions with cancer cells as well as in antigen presentation and the formation of adaptive immunity [20]. Although hypersialylation is a common feature found in cancer cells, sialoglycans can also be expressed on secreted glycoproteins, glycolipids, and the cell surface of immune cells [21, 22]. To investigate the potential interactions of Siglec receptors with ligands on the surface of MDSCs, we further assessed the sialylation pattern of MDSCs in the TME. Lectin staining was performed to assess surface sialoglycan ligands, including Sambucus Nigra Lectin (SNA), which detects α-2,6-linked sialic acids, and Maackia Amurensis Lectin II (MALII), which detects α-2,3-linked sialic acids. The staining intensity of PB-derived MDSCs from lung cancer patients was significantly greater for both lectins than for myeloid cells from healthy donors (Fig. 2A, B). No changes were detected in the levels of peanut agglutinin (PNA), a galactosyl (β-1,3) N-acetylgalactosamine structure that is usually masked by sialic acid binding (Fig. S2A). In addition, mass spectrometry (MS)-based analysis of released N-glycans from cancer patient-derived CD33^+^ cells revealed a clear increase in the abundance of terminally sialylated N-glycans containing multiple sialic acids compared to that in myeloid cells from healthy donors (Fig. 2C, D). The main N-glycan structures found on healthy donor CD33^+^ cells were core-fucosylated N-glycans with mono-sialic acid and PolyLacNAc. In addition, N-glycans containing more than 2 fucoses were observed, indicating the presence of Lewis structures.

We further studied the expression of ligands on the surface of murine myeloid cells using lectin staining. Similar to the findings observed in human MDSCs, increased levels of SNA were detected in both the CD11b^+^Ly6G^+^ and CD11b^+^Ly6C^+^ populations in the blood of tumor-bearing mice compared to their naïve littermates (Fig. 2E, F). No significant differences were observed in MALII or PNA levels (Fig. S2B–E). These data show the differential expression of sialoglycans on cancer-associated suppressive myeloid cells in humans and mice.

### Depletion of Siglec-E in myeloid cells prolongs survival

**Fig. 3:**
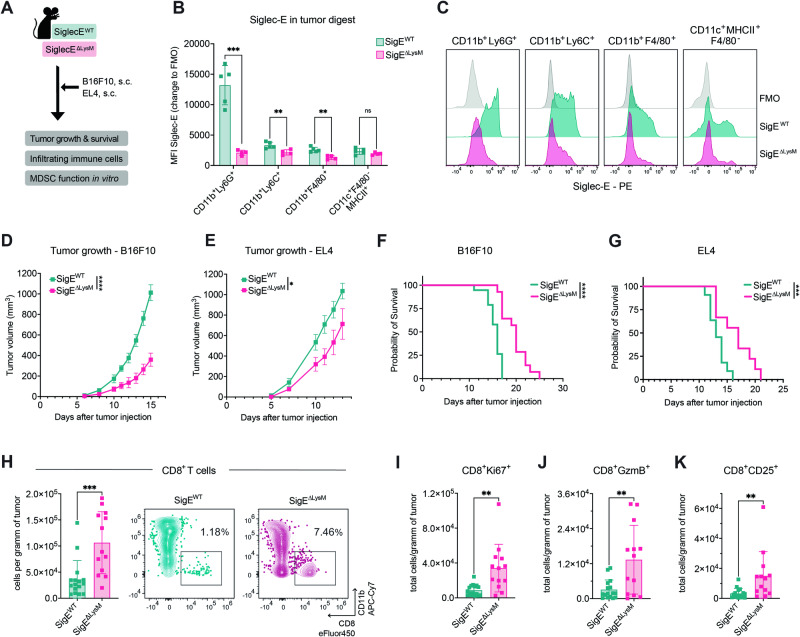
Siglec-E depletion on myeloid cells decreases tumor growth in mice. **A** Experimental setup: Siglec-ExLysM-Cre mice (SigE^ΔLysM^) and Siglec-E wild-type (SigE^WT^) littermates were subcutaneously injected with B16F10 or EL4 cells. Tumor growth, survival probability, tumor immune cell infiltration and suppressive capacity of Gr1^+^CD11b^+^ cells in vitro were analyzed. **B** The MFI of Siglec-E expression was assessed in myeloid cells in the tumor homogenates at the endpoint in SigE^ΔLysM^ mice and SigE^WT^ littermates. The MFI of Siglec-E is shown as the change in fluorescence relative to that of the control (FMO). The cell populations were identified as gMDSCs (CD45^+^CD11b^+^Ly6G^+^), mMDSCs (CD45^+^CD11b^+^Ly6C^+^), macrophages (CD45^+^CD11b^+^F4/80^+^), or dendritic cells (DCs) (CD45^+^CD11c^+^MHCII^+^F4/80^-^). *n* = *4–5 mice per group*. **C** Representative results showing Siglec-E staining of the cell populations depicted in (**B**) as a histogram. Siglec-E expression was assessed in SigE^ΔLysM^ (pink) and SigE^WT^ (green) littermates and compared to that in the FMO control (gray). **D** Tumor growth according to pooled data from mice subcutaneously injected with B10F10 and (**E**) EL4 cells. *n* = *9–12 mice per group*. **F** Kaplan‒Meier survival curves from pooled data of mice injected subcutaneously with B16F10 cells. *n* = *9–12 mice per group*. **G** Kaplan‒Meier survival curves from pooled data from 2 experiments in which cells were injected with EL4. *n* = *9 mice per group*. **H** B16F10 tumors at the endpoint (**D**) were digested and analyzed by flow cytometry. Intratumoral CD8^+^ cells (CD45^+^CD19^-^NKp46^-^CD3^+^CD8^+^), (**I**) Ki67^+^ CD8^+^ T cells, (**J**) Granzyme B^+^ CD8^+^ T cells^+^ (GzmB^+^), and (**K**) CD25^+^ CD8^+^ T cells were quantified as cells per gram of tumor at the endpoint of the experiment. Exemplary results for intratumoral CD8^+^ cells in SigE^ΔLysM^ (pink) and SigE^WT^ (green) littermates are shown on the right (**H**). *n* = *13–16 mice per group*. The data are presented as the mean ± SD or SEM (**D**, **E**). Two-tailed unpaired Student’s *t* test or multiple unpaired *t* tests (**B**) were used. For survival analysis, the log-rank test was used, followed by the Šidák correction for multiple comparisons. Tumor growth was compared by mixed-effects analysis followed by Bonferroni’s multiple comparisons test. *P < 0.05, **P < 0.01, ***P < 0.001, and ****P < 0.0001

To further investigate the role of Siglec-E in myeloid cells in the TME, Siglec-E^loxP^ mice were crossed with LysMCre mice to specifically target Siglec-E on LysM-expressing cells (SigE^ΔLysM^). Compared with their Siglec-E wild-type (SigE^WT^) littermates, SigE^ΔLysM^ mice were evaluated for tumor growth, survival, and immune infiltration upon subcutaneous tumor injection (Fig. 3A). As we focused on suppressive myeloid cells, models of cancer-induced emergency myelopoiesis, including B16F10 melanoma and EL4 lymphoma syngeneic tumor models, were used [23]. During emergency myelopoiesis, the number of myeloid cells rapidly increases, which leads to the accumulation of immature, suppressive cells, including MDSCs.

Subcutaneous injection of both tumor cell lines resulted in increased numbers of CD11b^+^Ly6G^+^ and CD11b^+^Ly6C^+^ cells in the spleens of tumor-bearing mice compared to those in the spleens of their naïve littermates (Fig. S3A, B). Additionally, high numbers of MDSCs were found within B16F10 and EL4 tumors, making them suitable models for studying the effect of Siglec-E in myeloid-driven tumors (Fig. S3C). To confirm the deletion of Siglec-E in our model, Siglec-E expression in myeloid cells from tumor homogenates and spleens of EL4 and B16F10 tumor-bearing mice was assessed via flow cytometry (Figs. 3B, C, S1D, E). Compared with those in SigE^WT^ mice, the expression of Siglec-E in CD11b^+^Ly6G^+^ cells (gMDSCs), CD11b^+^Ly6C^+^ cells (mMDSCs), and CD11b^+^F4/80^+^ macrophages in the tumor and spleen of SigE^ΔLysM^ mice was significantly decreased. Among the infiltrating immune cells within the tumor and spleen, Siglec-E was the most highly expressed on CD11b^+^Ly6G^+^ cells, followed by CD11b^+^Ly6C^+^ and CD11b^+^F4/80^+^ cells (Figs. 3B, C, S3D, E), as previously observed (Fig. 1E, F). Neither Siglec-F, Siglec-G nor Siglec-H expression on intratumoral CD11b^+^Ly6G^+^ or CD11b^+^Ly6C^+^ cells was affected in SigE^ΔLysM^ mice (Fig. S3F, G).

Deletion of Siglec-E in myeloid cells resulted in prolonged survival and decreased tumor growth in SigE^ΔLysM^ mice compared to those in SigE^WT^ littermate mice in the B16F10 and EL4 tumor models (Fig. 3D–G). We observed increased CD8^+^ T-cell infiltration in SigE^ΔLysM^ mice that was associated with the increased proliferation of T cells and expression of functional T-cell markers, including Granzyme B (GzmB), Ki67, and CD25 (Fig. 3H–K). To avoid cancer model-dependent effects, we confirmed our findings using EL4 lymphoma tumor cells. Increased CD8^+^ T-cell infiltration was also found in EL4 tumor-bearing mice, strengthening the importance of our findings (Fig. S3H–K). The numbers of myeloid cells within the tumor and spleen were not altered (Fig. S3L–O), suggesting a qualitative change rather than a quantitative change in myeloid cells upon Siglec-E depletion, which possibly resulted in a less suppressive TME leading to greater effector T-cell infiltration.

### Siglec-E and sialoglycans shape the immunosuppressive capacity of murine MDSCs

**Fig. 4:**
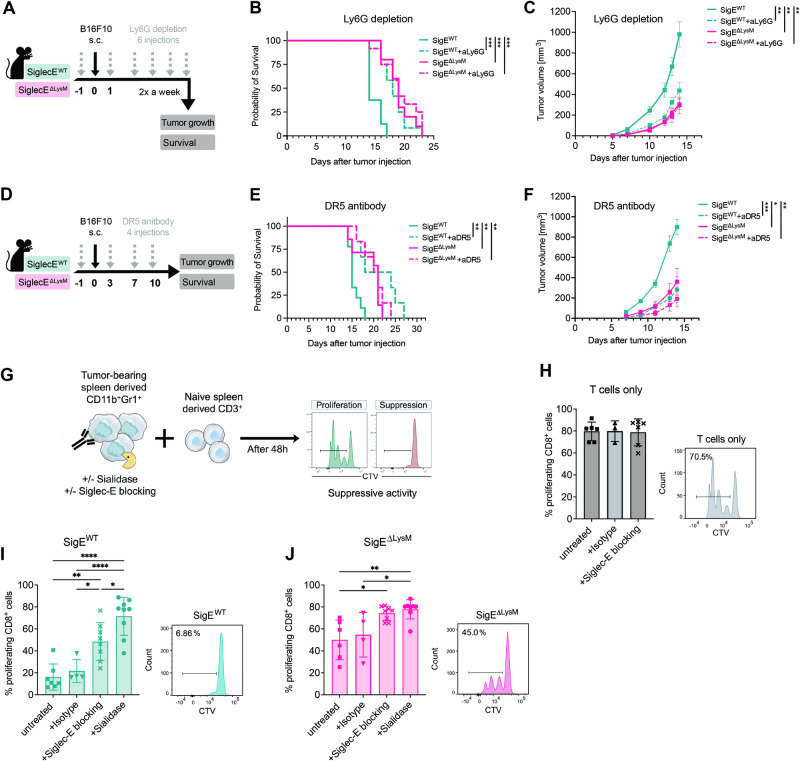
Reduced suppressive function of MDSCs lacking Siglec-E upon sialidase or Siglec-E blocking antibody treatment. **A** Experimental setup: Depletion of Ly6G-positive cells in SigE^ΔLysM^ mice and SigE^WT^ littermates bearing B16F10 tumors using a depleting antibody. Mice were injected up to 6 times (gray arrow) with the anti-Ly6G depletion antibody starting 1 day before subcutaneous B16F10 tumor injection (black arrow). Tumor growth and survival were monitored. **B** Kaplan‒Meier survival curves or (**C**) tumor growth curves from pooled experiments from (**A**). *n* = *9–11 mice per group*. **D** Experimental setup for assessing the effect of the DR5 antibody on SigE^ΔLysM^ mice and SigE^WT^ littermates subcutaneously injected with B16F10 tumors. **E** Kaplan‒Meier survival curves and (**F**) tumor growth curves from (**D**) with *n* = *6–7 mice per group*. **G** Experimental setup for assessing the suppressive effect of Gr1^+^CD11b^+^ (MDSC) cells on naïve CD3^+^ (T cell) cells. MDSCs were isolated from the spleens of B16F10 tumor-bearing SigE^ΔLysM^ mice and SigE^WT^ littermates. T cells were isolated from naïve littermates and stained with CellTrace Violet (CTV) to track T-cell proliferation by flow cytometry. Stained T cells were cocultured with MDSCs for 48 h in the presence of aCD3, aCD28 and IL-2. MDSCs were used immediately or pretreated with sialidase. A Siglec-E blocking antibody or rat IgG2a, κ isotype control was added to the cocultures as indicated. **H** Percentage of proliferating CD8^+^ T cells cocultured without MDSCs, (**I**) with MDSCs from SigE^WT^ mice or (**J**) MDSCs from SigE^ΔLysM^ mice. Exemplary results for each untreated condition are shown on the right, indicating untreated T cells alone (J, gray), SigE^WT^ (K, green), and SigE^ΔLysM^ (L, pink). *n* = *3–9 mice per condition*. The data are presented as the mean ± SD or SEM (**C**, **F**). Multiple paired *t* tests were used. For survival analysis, the log-rank test was used, followed by the Šidák correction for multiple comparisons. Tumor growth was compared by mixed-effects analysis followed by Bonferroni’s multiple comparisons test. *P < 0.05, **P < 0.01, ***P < 0.001, and ****P < 0.0001

To characterize the role of MDSCs and their suppressive function in our model, we performed MDSC depletion experiments. Considering that the highest expression of Siglec-E was found on gMDSCs, we hypothesized that Siglec-E depletion would mainly affect gMDSCs in our model. To test this hypothesis, Ly6G depletion was performed to eliminate Ly6G-expressing gMDSCs in SigE^ΔLysM^ mice and SigE^WT^ littermates upon B16F10 tumor cell injection (Fig. 4A). Depletion efficiency was validated in the blood of treated mice by gating on CD45^+^CD11b^+^Ly6C^intermediate^ cells to bypass antigen masking upon Ly6G depletion (Fig. S4A). The depletion of gMDSCs in SigE^WT^ mice prolonged their survival and tumor growth but did not affect the survival or tumor growth of SigE^ΔLysM^ mice lacking Siglec-E expression on myeloid cells (Fig. 4B, C). Similar results were observed upon treatment with a DR-5 agonist or Gr-1 depletion, in which both MDSC types were targeted via activation of TRAIL-mediated apoptosis or depletion of Ly6G- and Ly6C-expressing cells, respectively (Figs. 4D–F, S4B–D). Thus, Siglec-E expression on gMDSCs was likely involved in tumor progression in vivo. These results show that downregulating Siglec-E expression on myeloid cells, and in particular gMDSCs, inhibits tumor growth in different murine tumor models.

Although MDSCs are involved in various protumorigenic mechanisms, their ability to inhibit T-cell responses remains their key feature [18]. To test whether the suppressive function of MDSCs lacking Siglec-E is impaired, MDSCs were isolated by CD11b^+^Gr1^+^ negative selection from the spleens of B16F10 tumor-bearing mice, and their suppressive capacity was tested against proliferating naïve T cells in vitro (Fig. 4G). Stimulation of T cells with IL-2 and anti-CD3/28 antibodies led to high proliferation of T cells (Fig. 4H). MDSCs from SigE^WT^ mice strongly suppressed CD8^+^ T-cell proliferation, but SigE^ΔLysM^ MDSCs were significantly less suppressive, as shown by increased T-cell proliferation (Fig. 4I, J). This finding suggested that the reduced suppressive function of MDSCs lacking Siglec-E could generate a less suppressive TME in vivo, leading to increased T-cell infiltration and prolonged survival, as observed in SigE^ΔLysM^ mice.

To further evaluate whether suppressive MDSCs can be altered by interfering with the Siglec-sialoglycan axis, we added a Siglec-E blocking antibody to the cocultures or pretreated MDSCs with bacterial sialidase to reduce the levels of both α2,3- and α2,6-sialoglycans on the surface of the MDSCs. Using a Siglec-E blocking antibody, we decreased the suppressive effect of SigE^WT^ MDSCs, but no significant difference was observed after blocking Siglec-E on SigE^ΔLysM^ MDSCs or T cells alone (Fig. 4H–J). Pretreatment of MDSCs with sialidase strongly reduced the suppressive activity of both SigE^ΔLysM^- and SigE^WT^-derived MDSCs (Fig. 4I, J). These data indicate that sialoglycan ligands and Siglec-E on murine MDSCs are important players in the suppressive effect of MDSCs on murine T cells.

To further address the effect of sialidase treatment and the lack of Siglec-E in vivo, we generated B16F10 cells expressing viral sialidase (B16F10-sia) and injected them into mice to compare the tumor growth of SigE^ΔLysM^ mice to that of SigE^WT^ mice (Fig. S4E). B16F10-sia cells stably express membrane-bound viral sialidase, which cleaves α2,3- and α2,6-sialic acid from the surface of B16F10 cells and surrounding cells. Successful cell line generation was demonstrated by a decrease in MALII and SNA levels and the expression of sialidase in B16F10-sia cells compared to B16F10 wild-type cells (Fig. S4F). In accordance with the in vitro experiments, sialidase expression significantly decreased tumor growth in SigE^ΔLysM^ and SigE^WT^ mice compared to B16F10 wild type cells (Fig. S4G, H). In addition, SigE^ΔLysM^-treated mice injected with B16F10-sia cells showed the greatest improvement in survival, with 50% tumor-free survival. However, SigE^WT^ mice injected with B16F10-sia cells eventually reached the tumor endpoint. Analysis of intratumoral MDSCs revealed decreased numbers of CD11b^+^Ly6G^+^ infiltrating cells in B16F10-sia tumor-bearing mice from both SigE^ΔLysM^ and SigE^WT^ mice (Fig. S4I). No changes were observed in CD11b^+^Ly6C^+^ tumor-infiltrating cells (Fig. S4J).

To specifically assess the role of sialoglycan expression in cancer cells, we injected SigE^ΔLysM^ and SigE^WT^ mice with B16F10 and B16F10 GNE knockout (KO) tumor cells (Fig. S4K). B16F10 GNE KO cells lack UDP-N-acetylglucosamine 2-epimerase/N-acetylmannosamine kinase (GNE), a key enzyme involved in sialic acid biosynthesis, leading to decreased sialoglycan expression [24]. The successful generation of B16F10 GNE KO cells was confirmed by the downregulation of SNA in comparison to wild-type B16F10 cells (Fig. S4L). Injection of B16F10 GNE KO cells led to prolonged survival compared to that of B16F10 wild-type cells, as observed in previous publications with other tumor cell lines [25]. Tumor growth was not significantly different among SigE^ΔLysM^ and SigE^WT^ mice injected with B16F10 GNE KO cells and mice injected with the B16F10 wild-type cells, suggesting a Siglec-E-dependent effect in our model involving sialylated glycans on cancer cells (Fig. S4M–O). Taken together, these findings suggest that Siglec-E and sialoglycan ligands on murine MDSCs are involved in the suppression of CD8^+^ T cells. A reduction of sialoglycan ligands in the tumor microenvironment and on cancer cells is beneficial for controlling tumor size and increasing survival in vivo.

### Sialoglycans modulate the generation of human suppressive myeloid cells in vitro

**Fig. 5:**
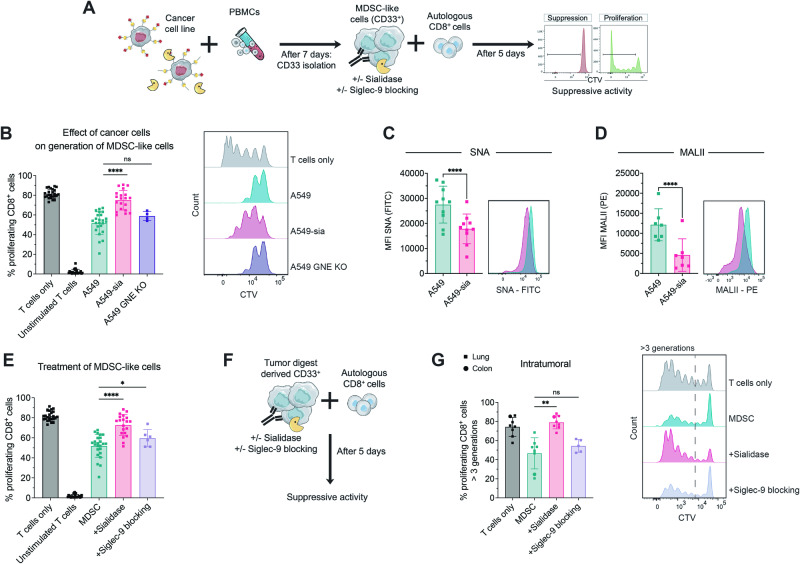
Targeting sialoglycans and Siglec-9 on suppressive human CD33^+^ cells attenuates their function. **A** Experimental setup for generating suppressive myeloid cells in vitro. Fresh PBMCs were isolated from buffy coats of healthy donors and cocultured with the indicated cancer cell lines at a ratio of 100:1. On Day 7, CD33^+^ cells were isolated by magnetic positive selection, and their suppressive effect on autologous CD8^+^ T cells was assessed. Suppressive CD33^+^ cells were immediately used or were pretreated with sialidase or a Siglec-9 blocking antibody as indicated. CD8^+^ T cells were stained with CellTrace Violet (CTV) and stimulated by the addition of IL-2 and anti-CD3/28 microbeads. After 5 days, CD8^+^ T-cell proliferation was assessed by FACS. **B** Percentage of proliferating CD8^+^ cells upon coculture with the indicated suppressive CD33^+^ cells. Suppressive myeloid cells were generated from A459, A549 cells stably expressing sialidase (A549-sia) or A549 GNE KO cancer cells. *N* = 4–24 donors. A representative histogram for each condition is shown on the right. **C** Lectin staining was performed on suppressive CD33^+^ cells on Day 7 of the experiment to assess SNA and (**D**) MALII levels. Representative images of A549 (green) and A549-sia-MDSC-like cells (pink) are shown on the right. *n* = *7–10 donors in paired conditions*. **E** Percentage of proliferating CD8^+^ cells upon coculture with suppressive CD33^+^ cells generated by A549 coculture. CD33^+^ cells were used immediately or were pretreated with sialidase or a Siglec-9 blocking antibody as indicated. *N* = *6–24 donors*. **F** Assay setup to test the suppressive capacity of tumor-digested CD33^+^ cells. CD33^+^ cells were freshly isolated from the tumor homogenates of lung or colon cancer patients and used immediately or were pretreated with sialidase or a Siglec-9 blocking antibody. CD8^+^ cells were isolated from fresh PBMCs from the same donor and stained with CTV. The suppressive activity was assessed on Day 5 by flow cytometry. **G** Percentage of proliferating CD8^+^ cells of more than 3 generations upon coculture with tumor-derived CD33^+^ cells. MDSCs were left untreated and pretreated with sialidase or a Siglec-9 blocking antibody. Exemplary results for each condition are shown on the right. *n* = *5–8 per condition*. The data are presented as the mean ± SD. A two-tailed paired *t* test was used. *P < 0.05, **P < 0.01, ***P < 0.001, and ****P < 0.0001

Next, we investigated whether targeting the Siglec-sialoglycan axis on human MDSCs affects their suppressive capacity. To test this hypothesis, we used an in vitro model to generate suppressive tumor-educated myeloid-derived CD33^+^ cells, which are subsequently referred to as MDSC-like cells, by adapting the protocol from Lechner et al. [26] (Fig. 5A). Using lung adenocarcinoma A549 and cervix adenocarcinoma HeLa cell lines, we generated highly immunosuppressive MDSC-like cells from fresh peripheral blood mononuclear cells (PBMCs) that were able to decrease CD8^+^ T-cell proliferation in an effector:target (E:T) ratio-dependent manner (Fig. S5A). This model was used to test the role of the Siglec-sialoglycan axis in MDSC generation and function via the suppression of autologous T cells as a functional readout, similar to the findings of our murine studies.

To determine the role of glycosylation of cancer cells during the generation of MDSC-like cells, we compared the ability of parental A549 cells, A549 cells expressing sialidase (A549-sia), and A549-GNE knockout (KO) cell lines to generate MDSC-like cells. A549-sia cells stably express membrane-bound viral sialidase, which cleaves α2,3- and α2,6-sialic acid from the surface of A549 cells and surrounding cells. To test sialidase activity, A549-sia cells were stained for lectins, which indicated effective desialylation via an increase in PNA and a decrease in SNA and MALII levels (Fig. S5B). Parental cancer cells as well as GNE-KO cells were able to generate MDSC-like cells with a strong suppressive phenotype (Fig. 5B). In contrast, coculture with sialidase-expressing cancer cells induced a significantly less suppressive phenotype, as shown by increased T-cell proliferation (Fig. 5B). To avoid a cell line-specific effect, we used HeLa and HeLa cells expressing sialidase (HeLa-sia) and observed similar results (Fig. S5D). To test the effect of sialidase expression on MDSC-like cells, we assessed the lectin levels in MDSC-like cells from A549-sia cocultures. Coculture with A549-sia cells led to desialylation of MDSC-like cells, as shown by a decrease in SNA and MALII levels as well as an increase in PNA compared to MDSC-like cells generated from parental cancer cell lines (Figs. 5C, D, S5C). These findings suggest that the level of sialoglycan ligands on MDSC-like cells is important for their suppressive effect on T cells and has a more relevant biological function than the level of sialoglycan ligands on the surface of cancer cells themselves according to our in vitro model. Our findings indicate the important role of sialoglycan ligands in shaping the suppressive function of MDSC-like cells.

### Sialidase treatment and blockade of Siglec-9 attenuate the suppressive activity of myeloid cells

Next, we aimed to address the effect of sialidase treatment and Siglec-9 blockade as a therapeutic approach for treating in vitro-generated human suppressive myeloid cells. To this end, we generated MDSC-like cells as described in the previous section and pretreated them with either bacterial or viral sialidase to cleave surface sialoglycan ligands or with a Siglec-9 blocking antibody (Figs. 5A–E, S5E). Sialidase pretreatment and blockade of Siglec-9 significantly decreased the suppressive effect of MDSC-like cells against autologous T cells (Figs. 5E, S5E). Successful desialylation of cells by sialidase treatment was demonstrated by a significant increase in PNA staining (Fig. S5F). Similar results were obtained using HeLa-generated MDSC-like cells (Fig. S5G).

To further corroborate our findings, we used cancer patient-derived CD33^+^ cells from primary tumor homogenates together with autologous CD8^+^ T cells isolated from PBMCs (Fig. 5F). The addition of tumor-derived CD33^+^ cells from colon and lung cancer patients significantly decreased the proliferation of CD8^+^ T cells (Fig. 5G). Pretreatment of suppressive myeloid cells with sialidase led to a significant reduction in their inhibitory effect against CD8^+^ T-cell proliferation (Fig. 5G). The addition of a Siglec-9 blocking antibody did not significantly affect the suppressive capacity of CD33^+^ cells, suggesting that other sialic acid-binding receptors, including other Siglec receptors, could be involved. Our experiments using human MDSC-like cells and intratumoral patient-derived suppressive CD33^+^ cells support our finding that the interactions of Siglec receptors with cell surface sialoglycan ligands can regulate the suppressive potential of MDSCs, which is an interesting target for attenuating the suppressive function of MDSCs.

### Sialoglycans modulate the expression of functional markers and cytokines at the RNA level

**Fig. 6:**
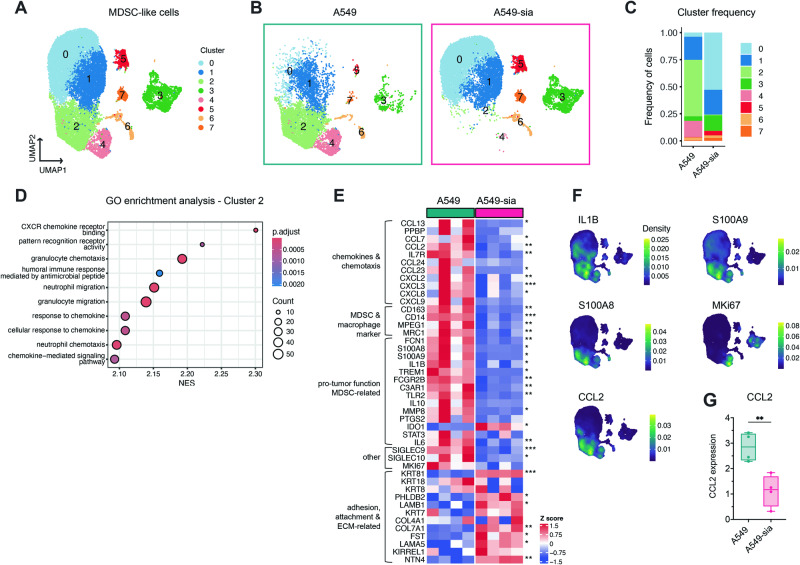
Desialylation of MDSCs downregulates MDSC functional markers and cytokines at the transcript level. Suppressive MDSC-like cells were generated in vitro by coculture with A549 or A549-sia cancer cell lines as described in Fig. 5. CD33^+^ cells were isolated on Day 7 and processed for single-cell RNA sequencing (scRNAseq). **A** Seurat analysis of the scRNAseq dataset projected in UMAP colored by cluster. *n* = *4 donors per treatment group*. **B** The dataset was subdivided into individual groups showing MDSC-like cells generated by A549 (green, left) or A549-sia (pink, right) coculture. **C** Stacked bar plots showing the frequency of each cluster annotated in (**A**) subclustered in A549- and A549-sia-generated MDSC-like cells. **D** Gene Ontology (GO) enrichment analysis of the top 10 upregulated gene sets found in Cluster 2. Dot plot showing the mean normalized enrichment score (NES) of the GO gene sets. The color coding indicates the adjusted p values, and the dot size is proportional to the gene count found in the listed pathway. **E** Heatmap of selected genes per patient divided into A549- and A549-sia-generated MDSC-like cells. The genes were functionally categorized into 5 groups: (i) chemokines and chemotaxis genes; (ii) MDSC and macrophage marker genes; (iii) protumor function MDSC genes; (iv) other genes; and (v) adhesion, attachment and ECM-related genes. **F** Gene expression of selected markers (IL1B, S100A9, S100A8, MKi67 and CCL2) as a density plot. The expression density is shown as a scale from blue (low) to yellow (high). **G** Expression of CCL2 was normalized to that of the donor.The data are presented as the mean ± SD. A two-tailed paired *t* test (**F**, **E**) was used. *P < 0.05, **P < 0.01, ***P < 0.001, and ****P < 0.0001

Next, we aimed to further dissect the underlying cellular and molecular mechanisms of the interaction between Siglec receptors and sialoglycan ligands on MDSCs. To better understand the differences in in vitro-generated MDSC-like cells and the role of sialoglycans in MDSC generation, transcriptomic analysis of MDSC-like cells generated with A549 and A549-sia cancer cell lines was performed by bulk RNA sequencing. Suppressive myeloid cells generated with A549-sia cells exhibited lower expression levels of various functional MDSC markers, protumor-function dMDSC-related genes and chemokine and chemotaxis molecules at the RNA level (Fig. S6A, B). Given the high complexity and plasticity of suppressive myeloid cells, we further investigated these differences via single-cell RNA sequencing (scRNA-seq). This approach provided more comprehensive information on individual cells and allowed us to dissect cellular heterogeneity and subpopulation gene expression (Figs. 6A, S6C). MDSC-like cells generated with A549 and A549-sia cancer cell lines showed a distinct composition of CD33+ cell populations (Fig. 6B, C). These changes were comparable between donors, indicating treatment-dependent clustering (Fig. S6D). Most prominently, suppressive MDSC-like cells generated by A549 coculture were enriched in Clusters 2 and 4, whereas fewer suppressive MDSC-like cells generated by A549-sia coculture were enriched in Clusters 0 and 3 (Figs. 6B, C, S6E). The MDSC-like cells generated from A549 cells not only were more suppressive in our in vitro assay (Fig. 5B) but also correlated significantly higher with a published MDSC-specific gene signature [27] than the MDSC-like cells obtained from A549-sia coculture (Fig. S6F). To better understand the underlying mechanisms of sialoglycan expression on MDSC-like cells, we performed pathway enrichment analysis across clusters (Fig. S6G). Most prominently, Cluster 2, consisting of highly suppressive MDSC-like cells, was associated with various pathways involving chemokines and chemotaxis molecules, highlighting its involvement in the suppressive function of MDSCs (Fig. 6D). The other highly populated cluster of MDSC-like cells generated by coculture with A549 cells, Cluster 4, was associated with cell division and proliferation pathways (Fig. S6G). To decipher the differences between MDSC-like cells generated in vitro, we analyzed (i) chemokines and chemotaxis, (ii) MDSCs and macrophage markers, (iii) protumor functions, and (iv) adhesion, attachment and ECM-related genes in MDSC-like cells generated from A549 and A549-sia cells (Fig. 6E). Many differentially expressed markers were already observed by bulk RNA sequencing (Fig. S6A). Various previously described MDSC protumor function-related genes, including S100A8/9, PTGS2, IL10, and IL1B, were significantly downregulated at the RNA level in less suppressive MDSC-like cells obtained by A549-sia coculture [18, 28]. Additionally, the MDSC-like cells generated from A549-sia cells exhibited significant downregulation of the expression of chemokine and chemotaxis molecules, including CCL2, CCL13, CXCL7, and CXCL2, at the RNA level and significant upregulation of many genes involved in adhesion and attachment. Important MDSC markers, including IL1B, S100A8 and S100A9, were primarily expressed by Cluster 2, underscoring the critical relevance of this cluster for the suppressive function of MDSC-like cells (Fig. 6F). In addition, Cluster 2 exhibited a significant increase in CCL2 expression. Higher expression of CCL2 across all donors was further observed in suppressive MDSC-like cells generated from A549 cells compared to A549-sia cells generated MDSC-like cells (Fig. 6G). Elevated expression of the proliferation marker Ki67 in Cluster 4 underlined the findings of the pathway enrichment analysis, identifying Cluster 4 as the proliferation cluster (Figs. 6F, S6G) [29]. In summary, the transcriptional analysis allowed to connect the decreased suppressive capacity of MDSC-like cells generated with A549-sia in vitro with a reduction of functional markers and chemokines on RNA level. Changes in the levels of sialoglycan ligands on MDSCs are involved in the transcriptional regulation of various important genes.

### Decreasing the number of sialoglycan ligands on MDSCs reduces immune-inhibitory CCL2 production and enhances anticancer immunity

**Fig. 7:**
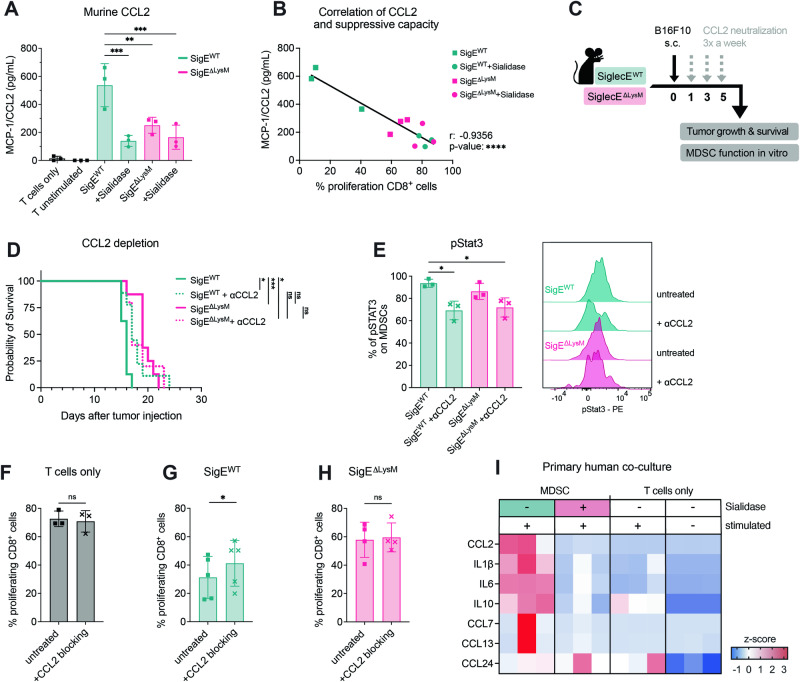
CCL2 is involved in T-cell suppression via the Siglec-sialoglycan axis in suppressive myeloid cells. **A** MCP-1/CCL2 found in the supernatant of murine MDSC:T-cell cocultures at the endpoint of the experiment from Fig. 4B–D. MDSCs were untreated, or were pretreated with sialidase or a Siglec-E blocking antibody*. n* = *3 donors per group*. **B** Correlation of MCP-1/CCL2 levels measured in supernatants of murine MDSC:T-cell cocultures at the endpoint and percentage of proliferating CD8^+^ T cells from the same conditions from (**A**). *n* = *3 donors per group*. **C** Experimental setup: Neutralization of CCL2 using a neutralization antibody in SigE^ΔLysM^ mice and SigE^WT^ littermates bearing B16F10 tumors. Mice were injected with a CCL2 neutralization antibody up to 3 times a week (gray arrow) starting 1 day after subcutaneous B16F10 tumor injection (black arrow). Tumor growth and survival were monitored, and the suppressive capacity of MDSCs was analyzed in vitro. **D** Kaplan‒Meier survival curves from pooled data from 2 independent experiments. *n* = *5–8 mice per group*. **E** B16F10 tumors at the endpoint (**C**) were digested, cocultured with pervanadate and analyzed by phospho-flow cytometry for phosphorylated STAT3 (pSTAT3). The percentage of pSTAT3 gated on total MDSCs is shown. Representative histograms for each condition are shown on the right. *n* = *3 mice per group*. **F** Suppressive capacity of MDSCs against naïve T cells. Percentage of proliferating CD8^+^ T cells cocultured without MDSCs, (**G**) with MDSCs from SigE^WT^ mice or (**H**) MDSCs from SigE^ΔLysM^ mice with or without addition of CCL2 blocking antibody. *N* = *3–5 mice from N* = *3 experiments*. **I** Cytokine expression found in the supernatant of human primary CD33^+^:CD8^+^ cell cocultures at the endpoint of the experiment from Fig. 5H. CD33^+^ cells were left untreated or pretreated with sialidase. *Z* scores were calculated for each cytokine and are shown on a color scale from blue (low) to red (high). *n* = *3 donors per group*. The data are presented as mean ± SD. Two-tailed paired *t* tests or unpaired *t* tests (**E**) were used. R shows the Pearson correlation coefficient. For survival analysis, the log-rank test was used, followed by the Šidák correction for multiple comparisons. *P < 0.05, **P < 0.01, ***P < 0.001, and ****P < 0.0001

Suppressive myeloid cells are involved in various protumorigenic mechanisms that can be mediated by the production of suppressive cytokines, including chemokines [30]. Given the changes in the RNA levels of various cytokines and chemokines, we wanted to further understand the impact of Siglec-sialoglycan interactions on the chemokine and cytokine production of MDSCs. To this end, we analyzed the cytokines and chemokines in MDSC-T-cell coculture supernatants by ELISA. By checking murine coculture supernatants (from Fig. 4J–L), we found various cytokines in cocultures compared to T cells alone, including CCL2, IL1β, IL-6, and IL-10 (Fig. S7A). CCL2 was highly increased in the supernatants of suppressive SigE^WT^ MDSCs compared to those in the SigE^ΔLysM-^ and sialidase-treated cells (Figs. 7A, S7A). Furthermore, the suppressive capacity of MDSCs was strongly correlated with the CCL2 level detected in the supernatant, indicating the relevant role of CCL2 in MDSC function (Fig. 7B).

CCL2 is widely described in the context of MDSCs and can act as a chemoattractant that is involved in the migration of myeloid cells and contributes to intratumoral MDSC accumulation [31]. Apart from its role as a chemoattractant, CCL2 facilitates the immunosuppression of T cells by regulating the suppressive functions of MDSCs via STAT3 in colorectal cancer [32] and is expressed not only by cancer cells but also by TAMs and MDSCs [33, 34]. To further evaluate the role of CCL2 as a mediator of MDSC suppression, the effect of CCL2 neutralization on tumor growth in SigE^ΔLysM^ and SigE^WT^ mice was examined (Fig. 7C). CCL2 neutralization in vivo led to prolonged survival in SigE^WT^ mice but did not significantly alter the survival of SigE^ΔLysM^ mice, indicating the involvement of Siglec-E signaling on suppressive myeloid cells (Fig. 7D). CCL2 depletion in vivo led to a decrease of phosphorylated STAT3 (pSTAT3) levels in tumor-infiltrating MDSCs, as described previously [32] (Fig. 7E). To test whether CCL2 was directly involved in the suppressive function of MDSCs, we analyzed the effect of a CCL2 blocking antibody on MDSC function against T cells in vitro (Fig. 7F–H). In accordance with the in vivo results, CCL2 blockade significantly decreased the suppressive effect of SigE^WT^ MDSCs (Fig. 7G) but did not impact the suppressive effect of SigE^ΔLysM^ MDSCs (Fig. 7H).

To further investigate the effect of sialidase treatment on chemokine and cytokine expression by human MDSCs, cytokine levels were measured in primary human cocultures from intratumoral suppressive myeloid cells (from Fig. 5G). As observed in mice, high levels of CCL2 were detected in the supernatants of suppressive myeloid cells, but pretreatment of primary human intratumoral CD33^+^ cells with sialidase significantly decreased CCL2 secretion (Figs. 7I, S7B). In addition, high levels of IL-1β, IL-6, and IL-10 were detected in suppressive CD33^+^ cell supernatants (Fig. 7I). Sialidase-treated CD33^+^ cells and T cells cultured alone exhibited low to no CCL2 expression. These results suggest that interactions between cell surface sialoglycan ligands and Siglec receptors promote the generation of suppressive myeloid cells that inhibit sufficient anticancer immunity by secreting CCL2.

## Discussion

Although the Siglec-sialoglycan axis is gaining attention as a potential glycoimmune checkpoint in cancer, little is known about the expression and effect of Siglec receptors and sialoglycan ligands on MDSCs [11]. Here, we showed that targeting cell surface sialoglycan ligands and Siglec receptors on MDSCs can decrease their suppressive capacity by downregulating cytokines and chemokines, mainly via CCL2.

Previous preclinical studies have demonstrated the anticancer effects of blocking Siglec receptors and/or sialidase treatment on various immune cell types, including T cells, NK cells, and myeloid cells, such as TAMs, resulting in a TME permissive to successful cancer immunotherapy [6, 10, 12, 13, 25, 35–38]. Here, we advance the understanding of how Siglec-sialoglycan interactions on suppressive myeloid cells can shape an immunosuppressive environment via the secretion of inhibitory CCL2 in the context of cancer across different human and murine models.

Although we observed a strong suppressive effect of Siglec-E deletion and blockade on myeloid cells in mice, blockade of a single inhibitory Siglec receptor on suppressive myeloid cells by a Siglec-9 blocking antibody resulted in a less pronounced effect in human cell culture models. This could be explained by the fact that compared to murine suppressive myeloid cells that mainly express Siglec-E in cancer, human suppressive myeloid cells express different, possibly redundant, inhibitory CD33-related Siglec receptors. It is possible that other Siglec family members, such as Siglec-5, Siglec-7, or Siglec-10, are involved in modulating the suppressive capacity of human MDSCs and that their importance might be variable or interchangeable between different tumors [13]. Sialidase treatment may be able to circumvent this effect by cleaving ligands for multiple Siglec receptors. A strong effect of sialidase pretreatment on the suppressive capacity of myeloid cells was observed across all of the assays, which supports this hypothesis. A first-in-human trial using a human bisialidase as a cancer immunotherapy against solid tumors showed tolerability and desialylation of immune cells in the peripheral blood [39] (NCT05259696). Additional work will be needed to investigate the effect of sialidase treatment on MDSCs in this setting, but it seems encouraging to modulate various players involved in generating a suppressive TME, including MDSCs and TAMs [12].

A strong effect on the suppressive capacity of MDSC-like cells was observed upon constant expression of sialidase by cancer cells, resulting in the desialylation of MDSC-like cells. Single-cell sequencing further linked the removal of sialoglycan ligands to the downregulation of functional MDSC markers and decreased chemokine and chemotaxis pathway activity at the transcriptional level. Similarly, the suppressive capacity of MDSCs was strongly reduced in human and murine MDSC:T-cell coculture models upon pretreatment of MDSCs with sialidase. Thus, sialoglycan ligands and Siglec expression on MDSCs are involved in shaping the suppressive capacity of MDSCs. These data indicate that interactions between Siglec receptors and sialoglycan ligands on myeloid cells play a role in shaping the suppressive function of MDSCs. The expression of *cis*-ligands on various immune cells has been described previously and proposed as a possible mechanism on MDSCs and immature DCs [17, 40]. However, further studies are needed to investigate whether Siglec receptors and sialoglycan ligands on MDSCs communicate in *cis* interaction on the same cell or *trans* interaction between neighboring MDSCs or cancer cells.

Most Siglec receptors are classified as inhibitory receptors that harbor tyrosine-based signaling motifs, such as ITIM domains, which can recruit and activate tyrosine phosphatases, including SHP-1 and SHP-2 [5]. We identified the interaction of sialoglycan ligands and Siglecs on MDSCs as a stimulus for MDSCs, which led to the release of suppressive cytokines (CCL2, IL-6, and IL-10). Blockade of this interaction resulted in decreased suppressive effects on T cells and downregulation of suppressive cytokines at the transcriptional level. In line with our findings, others have demonstrated the activation of macrophage and monocyte signaling upon Siglec-9 engagement via the MEK/ERK pathway [36]. In addition, the binding of CD33/Siglec-3 by the S100A9 family on MDSCs is involved in MDSC expansion and accumulation, resulting in the release of suppressive cytokines [41]. Therefore, the expression of Siglecs and sialoglycan ligands seems to be highly context-dependent and cell type-specific and can cause “classical” engagement and ITIM domain signaling, as well as a positive feedback loop that maintains the signaling of suppressive myeloid cells. However, the exact types of sialoglycan ligands involved are unclear, and further studies are needed to determine the exact mechanism by which sialoglycans can support the immunosuppressive properties of suppressive myeloid cells in cancer. Moreover, it remains unclear what role other lectins play after using sialidase and exposing lactosamine residues that could again bind to other immunomodulatory lectins, including galectins [42].

In addition, we identified CCL2 as an important immune-inhibitory chemokine released upon interactions between inhibitory Siglec receptors and sialoglycan ligands on suppressive myeloid cells. Previously, CCL2 was shown to impact the secretion of effector molecules and to contribute to T cell suppression via STAT3 signaling in MDSCs [31, 32]. Depletion of CCL2 in our in vivo model supported these findings, as evidenced by the downregulation of STAT3 upon CCL2 neutralization. Additionally, myeloid cells express high levels of CCR2, which is a promising target for interfering with MDSC migration to the TME, and can also express CCL2 themselves [14, 33, 34]. Therefore, it is not surprising that we found a strong association between the suppressive effect of MDSCs and CCL2 and that blocking CCL2 led to improved T cell proliferation. Nevertheless, we are the first to propose that the Siglec-sialoglycan axis on MDSCs and CCL2 expression are linked. In addition, CCL2 was highly expressed at the RNA level in suppressive MDSCs and coexpressed with functional MDSC markers. However, further investigations are needed to better understand the role of other cytokines involved, including IL-1β, IL-6 and IL-10.

Our study has some limitations. By using human CD33^+^ cells and a murine LysM-Cre model, we targeted a variety of myeloid cells, and it would be desirable to specifically target each of the suppressive myeloid cell subtypes to determine their individual contributions to immunosuppression. However, suppressive myeloid cells are closely related, and recent publications utilizing in-depth transcriptional, biochemical and phenotypical characterization have revealed the high complexity and plasticity of these cells [14, 27, 43, 44]. A clear distinction and definition by phenotype and the functional relevance of subtypes of myeloid cells are still lacking. Additionally, our assays focused on the suppressive effect of MDSCs against T cells, which is the gold standard [18]. The interaction between Siglec receptorss and sialoglycan ligands on MDSCs may also have additional functions on other immune cells, which need to be addressed.

Taken together, these findings indicate that cancer-associated suppressive myeloid cells express high levels of inhibitory Siglec receptors and sialoglycan ligands, inducing an immunosuppressive phenotype. We also identified CCL2 as a major inhibitory mediator of this effect. Blocking the Siglec-sialoglycan axis using sialidase or another broader approach targeting different Siglec receptors could render the immunosuppressive TME making it permissive for cancer immunotherapy such as immune checkpoint inhibition. Therefore, agents targeting sialoglycans or Siglec receptors could be used to treat cancers characterized by a high infiltration of suppressive myeloid cells.

## Materials and methods

### Patient samples

Tumor and blood samples were collected at the University Hospital Basel, and buffy coats from healthy donors were obtained from the blood bank of the University Hospital Basel, Switzerland. Sample collection and use of corresponding clinical data were approved by the local ethics committee in Basel, Switzerland (Ethikkommission Nordwestschweiz, EKNZ, Basel Stadt. Switzerland), and written informed consent was obtained from all the donors before sample collection. Healthy donors (HDs) were defined according to the inclusion criteria from the blood donation center in Basel. HD patients were 18–60 years old, had a minimal weight of 50 kilograms and were in good general health. The exclusion criteria included recent dental treatment (<14 days), tattooing, piercing or permanent makeup (<4 months), tick bites (<4 weeks), gastroscopy (<4 months), recent whole-blood donations (<3 months), surgery (<12 months), travel to certain countries with endemic diseases (up to 6 months), behavioral risk factors (sexual behavior), pregnancy and breastfeeding (1 year after giving birth). The donors were screened for infectious diseases, including HIV (AIDS), syphilis, hepatitis C and hepatitis B.

### Cell lines

HeLa and B16F10 cell lines were obtained from ATCC. A549, HeLa and EL4 cells were kindly provided by the Zippelius Laboratory, and HEK293T cells were kindly provided by the Bentires Laboratory, both of which were from the Department of Biomedicine, Basel. *The H1N1* viral sialidase-expressing cell lines A549-sia, HeLa-sia, and B16F10-sia as well as the EL4-GFP cell line were generated by lentiviral transduction as described below. A549-GNE KO and B16F10-GNE KO cells were generated as described before using CRISPR/CAS9 [25].

### Mouse strains

The experiments were performed in accordance with the Swiss federal regulations and approved by the local ethics committee of Basel-Stadt, Switzerland (Approval 3036 and 3099). All animals were bred in-house at the Department of Biomedicine Facility (University of Basel, Switzerland) in specific pathogen-free, ventilated HEPA-filtered cages under stable housing conditions of 45–65% humidity, a temperature of 21–25 °C, and a gradual light–dark cycle with light from 7:00 am to 5 pm. Mice were provided standard food and water without restriction (license: 1007-2H).

Siglec-E^loxP^ mice were generated in collaboration with Biocytogen Company, and LysM-Cre mice were generated as described previously [45]. To study the role of Siglec-E KO in LysM-Cre-expressing cells, Tm(Siglec-E x LysM-Cre) C57BL/6 mice were generated by crossing LysMCre mice with Siglec-E^loxP^ mice.

### Cell culture

Cell lines and primary cells were cultured at 37 °C and 5% CO_2_ and regularly checked for mycoplasma contamination. All of the cell lines except HEK293T cells were maintained in Dulbecco’s modified Eagle medium (DMEM, Sigma) supplemented with 10% heat-inactivated fetal bovine serum (FBS, PAA Laboratories), 1x MEM nonessential amino acid solution (Sigma) and 1% penicillin/streptomycin (Sigma).

All primary cells as well as HEK293T cells were maintained in Roswell Park Memorial Institute Medium (RPMI, Sigma) supplemented with 10% heat-inactivated fetal bovine serum (FBS, PAA Laboratories), 1x MEM nonessential amino acid solution (Sigma), 1 mM sodium pyruvate (Sigma), 0.05 mM 2-mercaptoethanol (Gibco) and 1% penicillin/streptomycin (Sigma).

### Tumor digestion and splenocyte and PBMC isolation

To obtain single-cell suspensions, human and mouse tumors were mechanically dissociated and subsequently enzymatically digested using accutase (PAA Laboratories), collagenase IV (Worthington), hyaluronidase (Sigma) and DNase type IV (Sigma) for 1 h at 37 °C under constant agitation. Afterward, the samples were filtered using a 70 µM cell strainer and washed. Precision counting beads (BioLegend) were added to all mouse tumors to calculate the number of cells per gram of tumor.

PBMCs were isolated from buffy coats by density gradient centrifugation using Hisopaque-1077 (Millipore) and SepMate PBMC isolation tubes (StemCell) according to the manufacturer’s protocol, followed by red blood cell lysis using RBC lysis buffer (eBioscience) for 2 min at RT. Subsequently, the cells were washed with PBS and ready for further analysis.

For splenocyte isolation, freshly harvested murine spleens were mechanically dissociated by filtering through a 100 µM filter. After washing, the red blood cells were lysed as described above.

For murine PBMC analysis, blood was collected from the tail vein of mice on Day 14 of the experiment via tail vein puncture. After washing, the red blood cells were lysed as described above, and the samples were immediately subjected to lectin staining.

Single-cell suspensions were used immediately or frozen for later analysis in liquid nitrogen (in 90% FBS and 10% DMSO).

### Tumor models

Siglec-ExLysM-Cre mice (SigE^ΔLysM^) were injected subcutaneously into the right flank with 500,000 B16F10 melanoma, B16F10-sia, B16F10-GNE KO, EL4 lymphoma or EL4 GFP cells in phenol red-free DMEM without additives. Sex-matched Siglec-E WT (SigE^WT^) littermates were used as controls. Mice were between 8 and 12 weeks of age at the beginning of the experiment, and conditional knockout was confirmed by genotyping and flow cytometry.

Tumor size was measured 3 times a week using a caliper. Animals were sacrificed before reaching a tumor volume of 1500 mm^3^ or when they reached an exclusion criterion (ulceration, severe weight loss, severe infection or bite wounds). Tumor volume was calculated according to the following formula: tumor volume (mm^3^) = (d^2^*D)/2, where D and d are the tumor length and width in mm, respectively.

### In vivo treatment

For in vivo Ly6G or Gr1 depletion, mice were injected intraperitoneally twice per week with 100 µg/mouse of anti-Ly6G depletion antibody (clone: 1A8, BioXCell) or 300 µg/mouse of anti-Gr1 depletion antibody (clone: RB6-8C5, BioXCell) in PBS. Antibody treatment began one day before tumor cell injection and was then administered twice a week up to 6 times per mouse.

For in vivo induction of TRAIL-mediated apoptosis, an anti-DR5 antibody (clone: MD5-1; BioXCell) was injected intraperitoneally twice per week. The first injection started 1 day before tumor injection, and the anti-DR5 antibody was administered up to 4 times per mouse.

For neutralization of CCL2 in vivo, mice were injected intraperitoneally 3 times a week with 200 µg/mouse of an anti-CCL2 neutralizing antibody (clone: 2H5, BioXCell) in PBS. Antibody treatment was started one day after tumor injection.

### Multiparameter flow cytometry

Multiparameter flow cytometry was performed on single-cell suspensions of the cell lines, PBMCs, splenocytes or tumor homogenates. To avoid nonspecific antibody binding, rat anti-mouse FcγIII/II receptor (CD16/CD32) blocking antibodies (BD Bioscience) were administered to murine cells and Fc receptor binding inhibitor polyclonal antibodies (Invitrogen) were administered to human cells. The cells were subsequently stained with live/dead cell exclusion dye (Zombie Dyes, BioLegend). Surface staining was performed with fluorophore-conjugated antibodies (Table S1) or with lectins for 30 min at 4 °C in FACS buffer (PBS, 2% FCS, 0.5 mM EDTA). The stained samples were fixed using IC fixation buffer (eBioscience) until further analysis. For intracellular staining, the cells were fixed and permeabilized using the Foxp3/transcription factor staining buffer set (eBioscience) and 1x permeabilization buffer (eBioscience) according to the manufacturer’s instructions. All of the antibodies were titrated to obtain the optimal signal-to-noise ratio. Antibody compensation was performed by staining primary cells or using an AbC Total Antibody Compensation Bead Kit (Invitrogen).

Data were acquired by an LSR II Fortessa (BD Biosciences), a CytoFLEX (Beckman Coulter) or a Cytek Aurora (Cytek Biosciences) flow cytometer and analyzed using FlowJo 10.8 (TreeStar, Inc.). Cell sorting was performed using a BD FACSAria III or BD FACSMelody (BD Bioscience). Doublets, cell debris and dead cells were excluded before performing downstream analysis. Fluorescence-minus-one (FMO) samples were used to define the gating strategy and calculate the mean fluorescence intensity (MFI).

To assess desialylation status, cells were stained with lectins as described above. The fluorophore-coupled lectins PNA-PE (GeneTex) and SNA-FITC (VectorBiolabs) and the biotinylated lectin MALII (VectorBiolabs) were used at a final concentration of 10 µg/mL. Biotinylated lectins were detected using PE-streptavidin (BioLegend). Sialidase was stained with neuraminidase antibody (LSBio) followed by PE-conjugated anti-rabbit IgG secondary staining (Invitrogen).

### Phospho-flow cytometry

Phospho-flow cytometry was used to assess the levels of phosphorylated STAT3 (pSTAT3). Frozen murine tumors were thawed, washed and cultured for 10 min with pervanadate at 37 °C in complete RPMI. Subsequently, the cells were stained for multiparameter flow cytometry as described above.

### In vitro generation of human suppressive myeloid cells

To generate suppressive human myeloid cells, we used an adapted version of the protocol established by Lechner et al. [26].

### A. Generation of MDSC-like cells

For in vitro MDSC induction, freshly isolated PBMCs from healthy donor buffy coats were cocultured for 7 days with different cancer cell lines (A549, A549-GNE KO, A549-sia, HeLa, or HeLa-sia) at a ratio of 1:100 in complete RPMI medium supplemented with 10 ng/mL GM-CSF (PeproTech). Cancer cells were seeded at an initial concentration of 1 × 10e4 cells/mL, and the same amount of medium supplemented with GM-CSF was added on Day 4 of the experiment. After one week, all confluent and adherent cells were collected using 0.05% trypsin-EDTA (Gibco).

### B. Isolation of MDSC-like cells

For MDSC isolation, CD33 positive cells were magnetically isolated using a human CD33 positive selection kit II (StemCell) following the manufacturer’s instructions. The isolated cells were resuspended in complete fresh RPMI medium for the suppression assay.

### C. Isolation of autologous CD8 T cells

Autologous CD8^+^ T cells were obtained from frozen PBMCs from the same donor using CD8^+^ microbeads from a human T-cell isolation kit (Miltenyi Biotec). To monitor cell proliferation, cells were labeled with 1.25 µM CellTrace Violet (Invitrogen) according to the manufacturer’s instructions. The washed cells were resuspended in complete RPMI and used for the suppression assay.

### D. Suppression assay

Isolated MDSC-like cells and autologous CD8^+^ T cells were cocultured at the indicated ratios for 5 days in a U-bottom plate in complete RPMI. T cells were stimulated by the addition of 100 IU/mL IL-2 (proleukin) and anti-CD3/CD28 stimulation using loaded MACSiBead particles (Miltenyi Biotec) at a ratio of 1:1 beads to cells. Unstimulated T cells and stimulated T cells without MDSC addition were used as controls. After five days, the supernatants were frozen at −80 °C, and the cells were stained for flow cytometry.

For Siglec-9 blocking, a Siglec-9 blocking antibody (Clone 191240, R&D Systems) was added at a final concentration of 10 µg/mL. For sialidase treatment, MDSCs were pretreated and washed before being added to the wells as described below.

### Human intratumoral-derived MDSC suppression assay

Fresh PBMCs and tumor homogenates were used immediately after isolation as described above. MDSCs were isolated from tumor homeogenates, and CD8 T cells were isolated from PBMCs using CD33 microbeads (Miltenyi) or CD8 microbeads (Miltenyi). The cells were cocultured at an MDSC:T-cell ratio of 1:4 in a U-bottom plate for 5 days in complete RPMI in the presence of 30 IU of IL-2 (proleukin) and human CD2/CD3/CD28 T-cell activator at a final concentration of 25 µL/mL (Immunocult, StemCell). For Siglec-9 blocking, a Siglec-9 blocking antibody (Clone 191240, R&D Systems) was added at a final concentration of 10 µg/mL. For sialidase treatment, MDSCs were pretreated and washed before being added to the wells as described below.

The supernatants were frozen at −80 °C, and the cells were stained for flow cytometry.

### Murine MDSC suppression assay

Murine T cells were enriched from wild-type mouse splenocytes by negative selection using a murine pan-T-cell isolation kit (EasySep, StemCell). To monitor T-cell proliferation, isolated T cells were stained with 2.5 µM CellTrace Violet (Invitrogen) according to the manufacturer’s instructions.

Murine MDSCs were isolated from the splenocytes of tumor-bearing mice by negative selection using a murine MDSC isolation kit (EasySep, StemCell). As indicated, the obtained MDSCs were used immediately or pretreated with sialidase as described below.

Isolated MDSCs and T cells were plated at a ratio of 1:1 in a 96-well flat bottom plate and cocultured for 48 h in complete RPMI in the presence of 50 IU of IL-2 (proleukin). For T-cell stimulation, the plate was coated with anti-CD3 (clone 17A2; BioLegend) and anti-CD28 (clone 37.51; BD Biosciences) antibodies. The supernatants were frozen at −80 °C, and the cells were stained for flow cytometry.

For Siglec-E blocking, purified anti-mouse Siglec-E antibody (M1305A02; BioLegend) or rat IgG2a or κ isotype control (BioLegend) was added at a final concentration of 10 µg/mL. CCL2 blocking was performed by the addition of 50 µg/mL CCL2 (Clone 2H5, BD).

### Sialidase treatment

To cleave terminal sialic acid residues, cells were treated with bacterial sialidase (*Vibrio cholerae*, Sigma) at a concentration of 10 µM for 20 min in PBS. Subsequently, the cells were washed with complete medium and used for downstream analysis. Additionally, viral sialidase (active *H1N1*; Sino Biological) and bacterial sialidase (*Arthrobacter ureafaciens*; Roche) were used for pretreatment, as indicated in the figure legends. If not stated otherwise, *Vibrio cholerae* bacterial sialidase was used.

### Lentivirus production and lentiviral transduction of cell lines

To generate cell lines expressing *H1N1* viral sialidase and GFP, A549, HeLa, B16F10 and EL4 cells were stably transduced with lentivirus.

For lentiviral production, 14 × 10e6 HEK293T cells were seeded 24 h before transfection in 18 mL of complete RPMI medium in a 15-cm culture dish. For the transfection mixture, 1.9 µg of pMD2. G, 3.5 µg of pCMVR8.74 and 5.4 µg of pLV transfer vector were mixed in 1.8 ml of jetOPTIMUS buffer (Polyplus). Then, 16.2 µl of jetOPTIMUS (Polyplus) was added to the prepared transfection mixture followed by 10 min of incubation. The medium was exchanged after 16 h, and the lentiviral particles were collected 24 and 48 h after medium exchange. The pooled supernatant was concentrated with 4x in-house-generated PEG-8000 solution and resuspended in PBS supplemented with 1% human serum albumin. Aliquots of the produced virus were stored at −80 °C until further use. pMD2. G and pCMVR8.74 were kindly provided by Didier Trono (Addgene plasmids #12259 and #22036).

For lentiviral transduction, 50,000 cancer cells were seeded in a 24-well plate in 500 µL of complete RPMI medium and allowed to rest overnight. The media was renewed by the addition of 100 µL of concentrated lentivirus and 8 µg/mL polybrene (Sigma). To increase transduction efficiency, spinoculation was performed, and the cells were centrifuged for 90 min at 800 × *g*. Afterward, the cells were incubated under standard cell culture conditions, and the transduction efficiency was frequently checked via flow cytometry staining to assess sialidase expression and PNA, MALII and SNA levels.

### Cytokine and chemokine analysis

The supernatants collected from murine and human cocultures were thawed on ice, and aliquots were subsequently sent on dry ice to Eve Technologies (Canada). Cytokine and chemokine concentrations were analyzed and calculated by Eve Technology. For visualization, normalized values (z scores) of each cytokine were calculated based on the mean and standard deviation of each marker.

### Bulk RNA sequencing

MDSC-like cells generated with A549 and A549-sia cancer cells from 4 different healthy donors were harvested after 7 days of coculture as described above. For purification of MDSCs, cells were stained with CD33-PE (Miltenyi) followed by CD33-positive selection using the EasySep Human PE Positive Selection Kit II (StemCell). To increase purity, the cells were further sorted for PE-positivity by an Aria III (BD Biosciences) flow cytometer. For RNA purification, the sorted cells were washed, and RNA was isolated using an RNeasy Plus Micro Kit (Qiagen), which included QIAshredder spin columns (Qiagen) for gDNA elimination.

Quality control (QC length profiling and concentration determination using RiboGreen) and library preparation (TruSeq stranded mRNA HT Kit by Illumina) were performed by the Genomics Core Facility of the University Basel. Sequencing was performed on four lanes of the Illumina NextSeq 500 instrument, resulting in 38 nt paired-end reads. The dataset was analyzed by the Bioinformatics Core Facility, Department of Biomedicine, University of Basel. cDNA reads were aligned to the ‘hg38’ genome using the Ensembl 104 gene models with the STAR tool (v2.7.10a) with default parameter values except for the following parameters: outFilterMultimapNmax=10, outSAMmultNmax=1, outSAMtype=BAM SortedByCoordinate, and outSAMunmapped=Within. At least 40 M read pairs were mapped per sample. The software R (v4.1.1) and the tool featureCounts from the Subread (v2.0.1) package from Bioconductor (v3.14) were used to count aligned reads per gene with default parameters except for -O, -M, --read2pos = 5, --primary, -s 2, -p, and -B. Further analysis steps were performed using R (v4.2.0) and multiple packages from Bioconductor (v3.15). The package edgeR (v3.38.1) was used to perform differential expression analysis. A gene was included in the analysis only if it had at least 1 count per million (CPM) in at least four samples. Gene set enrichment analysis was performed using a tool camera from the edgeR package and the Gene Ontology gene set (category C5) from MSigDB (v7.5.1). The raw sequences (FASTQ files) are available through the European Genome-Phenome Archive under accession number EGAS00001007618.

### Single-cell RNA sequencing

A549 and A549-sia MDSC-like cells from 4 different healthy donors were generated and processed by fluorescence-activated cell sorting as described for bulk RNA sequencing (see above). Single-cell RNA sequencing was performed using Chromium Next GEM Single-cell 3′ with feature barcode technology from 10x Genomics according to the manufacturer’s instructions. Approximately 20,000 cells were loaded in a 10x Genomics cartridge for target recovery. Cell-barcoded 3′ gene expression libraries were sequenced on an Illumina NovaSeq 6000 system and mapped to the GRCh38 human reference genome using CellRanger v. 7.1 (10x Genomics).

For single-cell demultiplexing, we utilized patient-specific single nucleotide polymorphisms (SNPs). To genotype Bi-Allelic SNPs on single cells, Cellsnp-lite [46] was used on the output bam files and cell barcodes derived from CellRanger. The SNP list hapmap_3.3.hg38_maf.vcf of the Genome Analysis Toolkit from the Broad Institute was utilized as a SNP reference. The generated output was subsequently processed with vireo [47] to assign the identified SNPs to the 4 individuals and annotate the resulting doublets. Identified donors were matched between wells based on SNP overlap and corresponding cells were analyzed by the Seurat R package. The sequences are available through the European Genome-Phenome Archive under accession number EGAS00001007618.

### Analysis of scRNA-seq data

The read count matrices were processed and analyzed in R v. 4.3.0 using Seurat v.5.0.1 [29] with default parameters for all functions, unless otherwise specified. Cell quality was investigated, and doublets and low-quality cells were removed based on the following criteria: nFeatures under 600, nCounts under 900 and a percentage of mitochondrial genes greater than 10%. Subsequently, the data were normalized to the variable features identified and scaled. After principal component analysis (PCA) reduction, the first 10 PCs were used for nearest neighbor analysis with a resolution of 0.4 for cluster identification. UMAP was also calculated for the first 10 PCs. Clusters were investigated, and those with no CD33 expression were identified as impurities and excluded from further analysis. Normalization, variable feature identification and dimensional reduction were repeated for the subset by using the first 16 PCs. A resolution of 0.2 was used, and the Leiden algorithm was used for cluster identification.

Gene set enrichment analysis was performed using the Seurat FindAllMarkers function, allowing the identification of up- and downregulated markers per cluster. The logfc.threshold was set to zero. Gene set enrichment analysis (GSEA) was performed for each cluster using the clusterprofiler package [48]. The results were ordered based on the normalized enrichment score, and only the highest enriched Gene Ontology terms were used for data interpretation.

### N-glycomic profiling

For N-glycomic profiling, harvested cells were extracted with lysis buffer containing 7 M urea, 2 M thiourea, and 10 mM dithioerythreitol in 40 mM Tris buffer supplemented with 1% protease inhibitor (Roche). The cell membranes were disrupted by high-intensity focused ultrasound (HICUS) with 10 cycles of 10 s of sonication at 16× magnification and 1 min on ice in between sonication and subsequent shaking for 4 h in a cold room. The protein extracts were alkylated with 100 mM iodoacetamide in the dark for 4 h at 37 °C. Ice-cold trichloroacetic acid was added to a final concentration of 10% w/v, and the mixture was left for one hour. After centrifugation at 20,000 × *g* for 30 min at 4 °C, the precipitated sample pellets were washed twice with ice-cold acetone and then lyophilized. The dry protein pellets were redissolved in 50 mM ammonium bicarbonate buffer (pH 8.5), 250 units of benzonase nuclease (Sigma‒Aldrich) were added, and the mixture was incubated for 30 min at 37 °C, followed by trypsin digestion overnight. After the activity of trypsin was deactivated, the protein mixtures were further treated with PNGaseF (New England Biolab). The released glycans were removed according to previous methods [49].

For MALDI-MS analyses, the glycan samples were permethylated using the sodium hydroxide/dimethyl sulfoxide slurry method, as described by Dell et al. [50]. The samples were dissolved in 20 µL of acetonitrile. One microliter of sample mixed with 10 mg/mL 2,5-dihydroxybenzoic acid (Bruker) in 70% acetonitrile with 1 mM sodium chloride was added to a MALDI target plate and analyzed by a Bruker RapiFlex^TM^ MALDI-TOF-TOF instrument. Permethylated high-mannose N-glycans and glycans from fetuin were used to calibrate the instrument prior to the measurement. The laser energy for each analysis was fixed, and the data were accumulated from 10 000 shots. The data were analyzed by GlycoWorkbench [51] and inspected manually. For relative quantification, the data were first deisotoped, and the peak height was used for the calculation based on the following equation:

$${Percentage}\,{of}\,{groupped}\,{structures}=\frac{{sum}\,{of}\,{peak}\,{height}\,{from}\,{groupped}\,{structures}}{{sum}\,{of}\,{peak}\,{height}\,{from}\,{all}\,{structures}}\times 100 \%$$

### Statistical analysis

All of the statistical analyses were performed using GraphPad Prism 9 or base functions in R v. 4.3.0. The statistical tests used and sample sizes are indicated in the figure legends.

*p* values > 0.05 were considered not significant, and *p* values < 0.05 were considered significant. Asterisks indicate the following: *p value < 0.05, **p value < 0.01, ***p value < 0.001, ****p value < 0.0001. *n* indicates the number of biological replicates. All of the bars within the graphs represent mean values, and the error bars represent standard errors of the mean (SEM) or standard deviation (SD) as indicated.

### Supplementary information

Figure S1

Figure S2

Figure S3

Figure S4

Figure S5

Figure S6

Figure S7

Supplementary Figure

Supplementary Table
