# Supplementary figures and images for: Engagement of sialylated glycans with Siglec receptors on suppressive myeloid cells inhibits anticancer immunity via CCL2
Source: Cell Mol Immunol. 2024 Mar 6;21(5):495–509. doi: 10.1038/s41423-024-01142-0 (PMC11061307; doi:10.1038/s41423-024-01142-0)

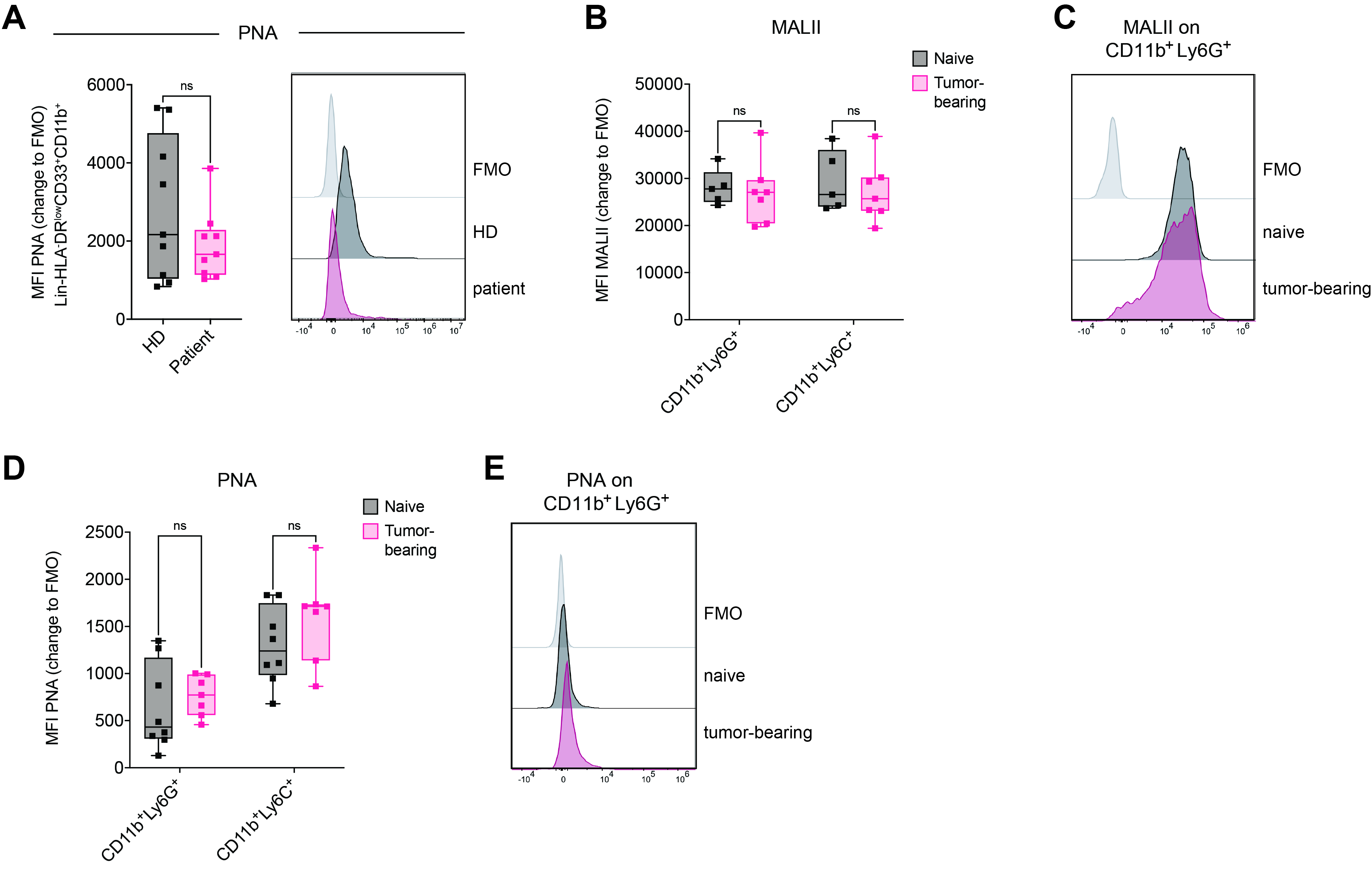

Supplement: Supplementary file 2 — Figure S2 [file 41423_2024_1142_MOESM2_ESM.tif]

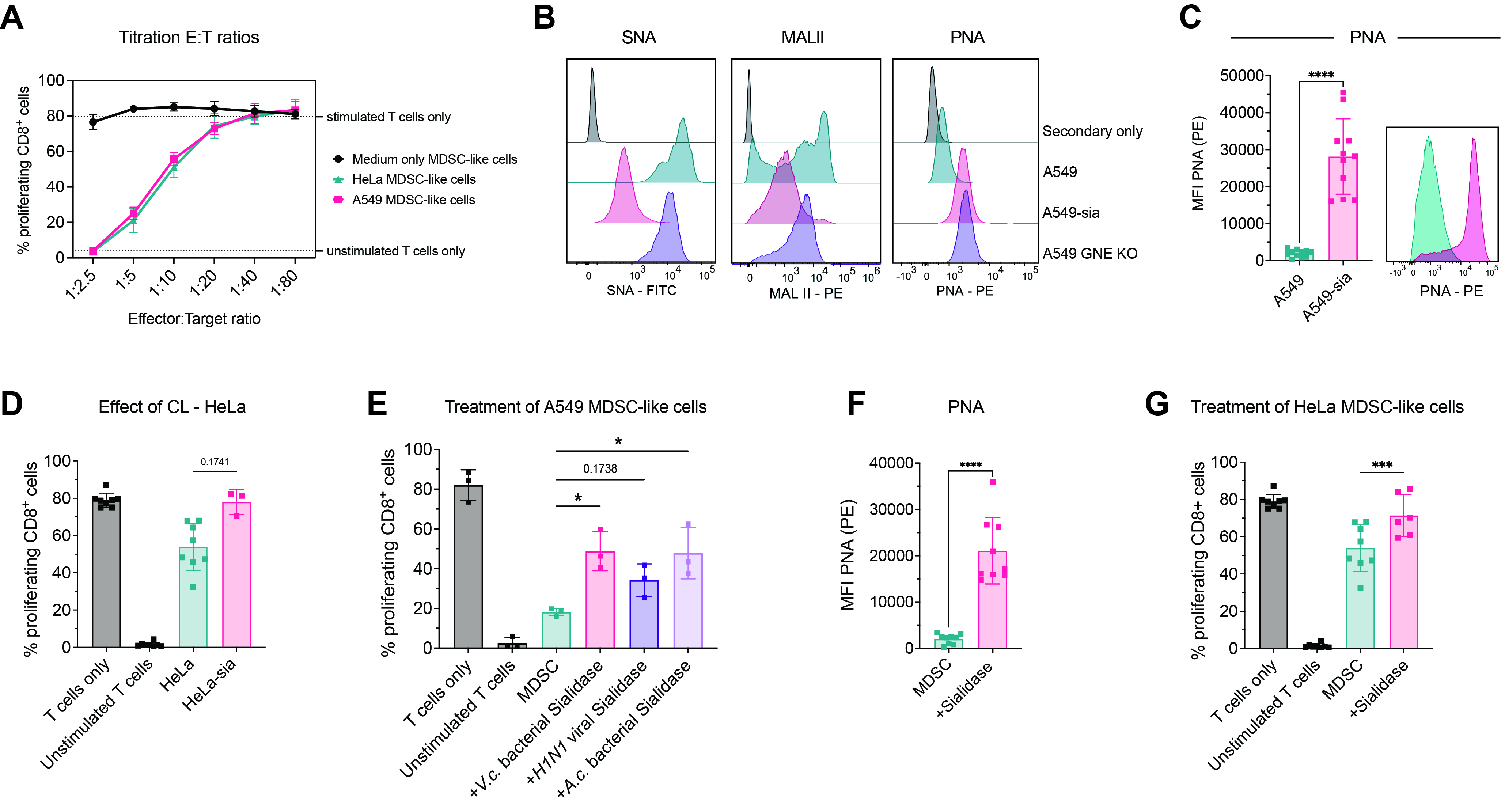

Supplement: Supplementary file 5 — Figure S5 [file 41423_2024_1142_MOESM5_ESM.tif]

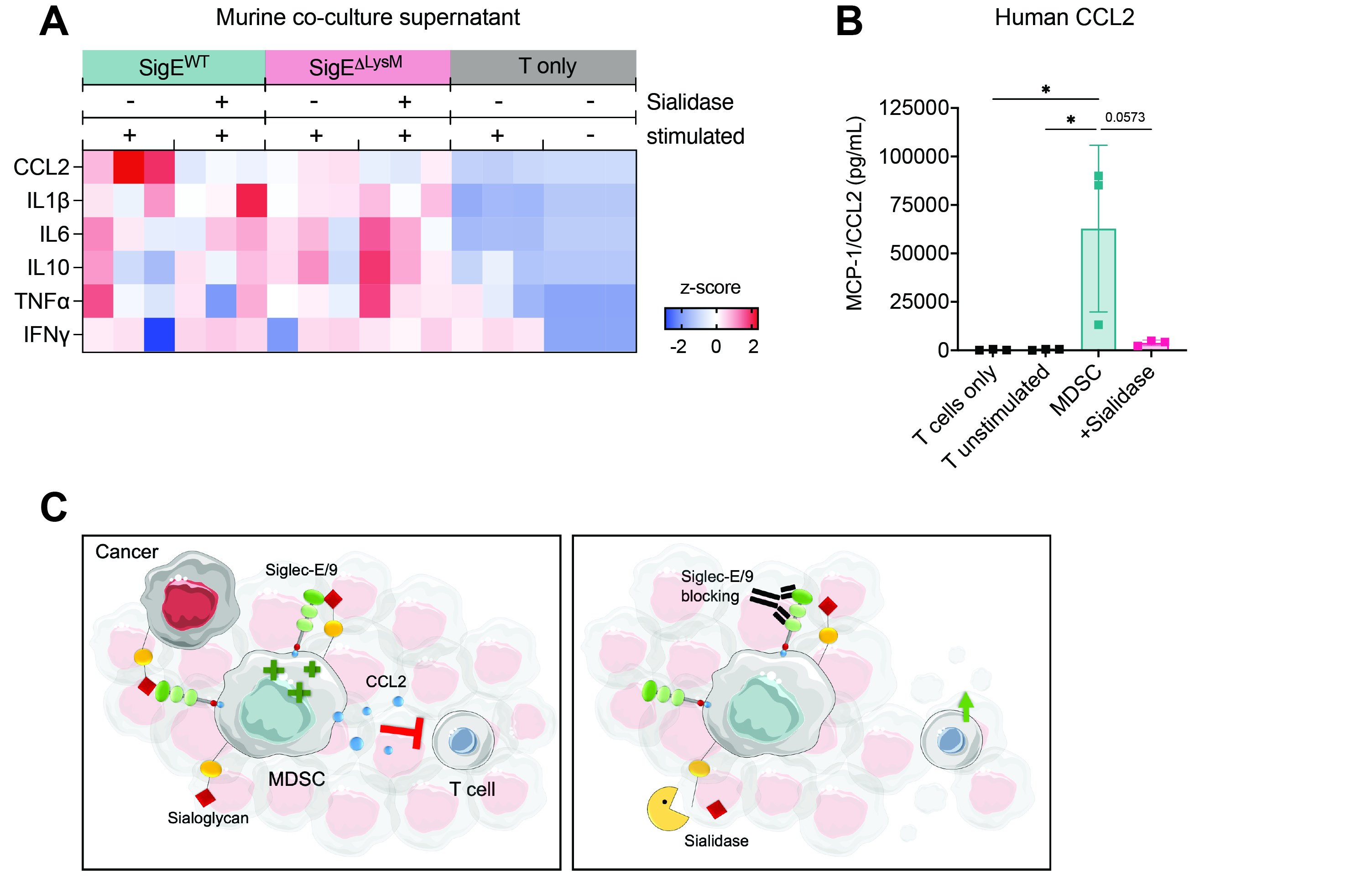

Supplement: Supplementary file 7 — Figure S7 [file 41423_2024_1142_MOESM7_ESM.tif]
