# Supplementary material for: Engagement of sialylated glycans with Siglec receptors on suppressive myeloid cells inhibits anticancer immunity via CCL2
Source: Cell Mol Immunol. 2024 Mar 6;21(5):495–509. doi: 10.1038/s41423-024-01142-0 (PMC11061307; doi:10.1038/s41423-024-01142-0)
Supplement: Supplementary file 8 — Supplementary Figure [file 41423_2024_1142_MOESM8_ESM.docx]

## **Fig. S1: Expression of Siglecs on myeloid cells from various origins**

**(A)** Exemplary gating strategy used for Fig. 1A-E. MDSCs were gated as CD45^+^Lin^-^(CD3^-^CD16^-^CD19^-^CD20^-^CD56^-^) HLA-DR^low^CD33^+^CD11b^+^ cells. Subpopulations of MDSCs were identified by CD14 and CD15 expression. The gating strategy used to assess Siglec receptors is shown. Gates were set according to fluorescence minus one (FMO) controls. **(B)** Percentages of Siglec-5, Siglec-7, Siglec-9 and Siglec-10 expressed on CD45^+^Lin^-^HLADR^low^CD33^+^CD11b^+^ cells derived from healthy donor peripheral blood (PB) or **(C)** intratumorally from lung cancer tumor digestion. *N=3-8 donors with at least N=2.* **(D)** Mean fluorescence intensity (MFI) of Siglec-5 and **(E)** Siglec-7 gated on CD45^+^Lin^-^HLADR^low^CD33^+^CD11b^+^ cells derived from healthy donors and from the PB of lung cancer patients. The MFI is shown as the change in FMO and was determined by flow cytometry. *n=7-9 donors per group.* **(F)** Representative gating strategy for identifying murine immune cell types in the tumor digest, peripheral blood and spleen. **(G)** Subcutaneously injected endpoint tumors from EL4 lymphoma engrafted mice were harvested and digested, and immune cell infiltration was assessed via multiparameter flow cytometry. Siglec-E, Siglec-F and Siglec-G expression was assessed in CD45^+^CD11b^+^Ly6G^+^ and CD45^+^CD11b^+^Ly6C cells. *n=5 mice per group.* **(H)** Spleens from naïve and EL4 lymphoma tumor-bearing mice at the endpoint were collected and analyzed for Siglec-E expression via flow cytometry. *n=3-8 mice per group*.

The data are presented as the mean ± SD. Two-tailed unpaired Student’s t test or multiple unpaired t tests (H) were used. *P<0.05, **P<0.01, ***P<0.001, and ****P<0.0001.

## **Fig. S2: Sialoglycan expression on myeloid cells in humans and mice**

**(A)** PNAs gated on peripheral blood (PB)-derived CD45^+^Lin^-^HLADR^low^CD33^+^CD11b^+^ cells from primary lung cancer patients and healthy controls. The MFI is shown as the change in FMO and was determined by flow cytometry. A representative histogram for each condition is shown on the right. *n=9 donors per group*. **(B, C)** Fresh blood from B16F10 tumor-bearing mice and naïve wild-type mice was collected on Day 14 after tumor inoculation and analyzed for MALII or **(D, E)** PNA gated on CD45^+^CD11b^+^Ly6C^+^ or CD45^+^CD11b^+^Ly6G^+^ cells. Exemplary results for each condition, including an FMO control, are shown on the right. The MFI is shown as the change in FMO. *5-8 mice per group.*

The data are presented as the mean ± SD Two-tailed unpaired Student’s t test or multiple unpaired t tests (B, D) were used. *P<0.05, **P<0.01, ***P<0.001, and ****P<0.0001.

## **Fig. S3: Siglec-E depletion in myeloid cells leads to survival benefits in emergency myelopoiesis tumor model mice**

**(A)** Spleens from naïve mice and from EL4 lymphoma or B16F10 melanoma tumor-bearing mice at the endpoint were collected and analyzed for CD11b^+^Ly6G^+^ and **(B)** CD11b^+^Ly6C^+^ cell infiltration. Exemplary flow cytometry plots for the groups are shown on the right. *n=5-9 mice per group*. **(C)** Tumor homeogenates were analyzed at the endpoint of myeloid cell infiltration. gMDSCs (CD45^+^CD11b^+^Ly6G^+^), mMDSCs (CD45^+^CD11b^+^Ly6C^+^), macrophages (CD45^+^CD11b^+^F4/80^+^) and dendritic cells (DCs) (CD45^+^CD11c^+^MHCII^+^F4/80^-^) are shown as percentages of CD45^+^ cells. *n=9-16 mice per group*. **(D)** The MFI of Siglec-E expression in B16F10 tumor-bearing mice was analyzed in myeloid cells in the spleen at the endpoint of the experiment. Representative results showing Siglec-E expression in select cell populations. Siglec-E was assessed in SigE^ΔLysM^ (pink) and SigE^WT^ (green) littermates and compared to that in the FMO control (gray). *n=6-9 mice per group*. **(E)** The MFI of Siglec-E expression in myeloid cells in the tumor homogenate was analyzed in EL4 tumor-bearing mice at the endpoint*. n=4-5 mice per group*. **(F)** Siglec-E, Siglec-F, Siglec-G and Siglec-H expression was assessed in mMDSCs (CD45^+^CD11b^+^Ly6C^+^) and **(G)** gMDSCs (CD45^+^CD11b^+^Ly6G^+^) from B16F10 tumor-bearing mice. *n=5-7 mice per group*. **(H)** Subcutaneous EL4-GFP tumors were analyzed at the endpoint via flow cytometry. Intratumoral CD8+ T cells at the endpoint (CD45^+^aliveCD19^-^NKp46^-^CD3^+^CD8^+^) were further subgated on **(I)** Ki67^+^ cells, **(J)** Granzyme B+ cells (GzmB^+^) and **(K)** CD25^+^ CD8+ T cells and quantified as the percentage of CD45^+^ cells*. n=4-5 mice per group*. **(L)** Tumor digestion from B16F10 tumor-bearing mice was analyzed at the endpoint of myeloid cell infiltration. gMDSCs (CD45^+^CD11b^+^Ly6G^+^) and **(M)** mMDSCs (CD45^+^CD11b^+^Ly6C^+^) were quantified as cells per gram of tumor. *n=9-12 mice per group.* **(N)** Spleens from B16F10 melanoma tumor-bearing mice at the endpoint were collected and analyzed for CD11b^+^Ly6G^+^ and **(O)** CD11b^+^Ly6C^+^ cells. The cells were quantified as the percentage of live CD45^+^ cells. *n=6-9 mice per group.*

The data are presented as the mean ± SD. Two-tailed unpaired Student’s t test, multiple unpaired t tests (D, E, F, G) or one-way ANOVA followed by Dunnett’s multiple comparisons test (A, B) were used. *P<0.05, **P<0.01, ***P<0.001, and ****P<0.0001.

## **Fig. S4: The combination** **of sialidase expression and a lack of Siglec-E on myeloid cells prolongs survival *in vivo***

**(A)** Representative results showing the depletion efficiency of the Ly6G depletion antibody in the blood of untreated and anti-Ly6G-treated mice. GMDSCs were assessed by conventional gating of CD45^+^ CD11b+Ly6G+ cells (left) as well as alternative gating on CD45^+^CD11b^+^Ly6C^intermediate^ cells to circumvent antigen masking. **(B)** Experimental setup: Depletion of Gr1-positive cells in SigE^ΔLysM^ mice and SigE^WT^ littermates bearing B16F10 tumors using a depleting antibody. Mice were injected up to 6 times (gray arrow) with a Gr1-depletion antibody starting 1 day before subcutaneous B16F10 tumor injection (black arrow). Tumor growth and survival were monitored. **(C)** Kaplan‒Meier survival curves or **(D)** tumor growth curves from pooled experiments from **(B)**. *n=10-13 mice per group*. **(E)** Experimental setup: SigE^ΔLysM^ and SigE^WT^ littermates were subcutaneously injected with B16F10 or B16F10 cells expressing sialidase (B16F10-sia). Tumor growth and the probability of survival were addressed as the main readouts. **(F)** B16F10 and B16F10-sia cells were stained for SNA, MALII and sialidase to validate the successful generation of stable cell lines. The cell lines were stained before each experiment, and representative results are shown. **(G)** Tumor volume and **(H)** Kaplan‒Meier survival curves from **(E)**. *n=8-12 mice per group.* **(I)** B16F10 tumors at the endpoint **(E)** were digested and analyzed by flow cytometry. Intratumoral gMDSCs (CD45^+^CD11b^+^Ly6G^+^) and **(J)** mMDSCs (CD45^+^CD11b^+^Ly6C^+^) are shown as percentages of CD45+ cells. *n=3-5 mice per group.* **(K)** Experimental setup: SigE^ΔLysM^ mice and SigE^WT^ littermates were subcutaneously injected with B16F10 wild-type or B16F10-GNE knockout (KO) cells. **(L)** B16F10 and B16F10-GNE-KO cells were stained for SNA to validate the successful generation of stable cell lines. **(M)** Tumor growth, **(N)** tumor growth on Day 12 after tumor injection and **(O)** probability of survival are shown as Kaplan‒Meier survival curves for the mice injected according to **(K)**. *n=5-6 mice per group*.

The data are presented as the mean ± SD or SEM (D, G, M). Tumor growth was compared by mixed-effects analysis followed by Bonferroni’s multiple comparisons test. An unpaired Student’s t test was used. For survival analysis, the log-rank test was used, followed by the Šidák correction for multiple comparisons. *P<0.05, **P<0.01, ***P<0.001, and ****P<0.0001.

## **Fig. S5: Sialylation modulates the suppressive potential of human CD33^+^ cells**

**(A)** Determination of the E:T ratio to validate the suppressive effect of CD33^+^ cells on CD8^+^ cells. Suppressive myeloid cells were generated by coculture with A549 (pink) or HeLa (green) cells or without cancer cells (black). The dotted lines indicate the proliferation of T cells alone with/without stimulation by IL-2 or aCD3/28 microbeads. *N=4 donors of N=2 experiments*. **(B)** A549, A549-expressing sialidase (A549-sia) and A549 GNE-KO cells were stained for SNA, MALII and PNA to validate the successful generation of stable cell lines. The cell lines were stained before each experiment, and representative results are shown. **(C)** PNA staining was performed on suppressive CD33^+^ cells generated from A549 or A549-sia cocultures on Day 7 of the experiment. *N=11 donors.* **(D)** Percentage of proliferating CD8^+^ cells upon coculture (1:10 ratio) with the indicated suppressive CD33^+^ cells. Suppressive myeloid cells were generated using HeLa or HeLa-expressing sialidase (HeLa-sia) cancer cell lines. *N=3-8 donors.* **(E)** Percentage of proliferating CD8^+^ cells cocultured with suppressive CD33^+^ cells generated by A549 cell coculture at a ratio of 1:5. CD33^+^ cells were used immediately or were pretreated with the indicated sialidases. *n=3 donors per group.* **(F)** Images of untreated A549-generated MDSC-like cells or sialidase-pretreated cells after 7 days. *N=9 donors.* **(G)** Percentage of proliferating CD8^+^ cells upon coculture (1:10 ratio) with suppressive CD33^+^ cells generated by HeLa cell coculture. CD33^+^ cells were used immediately or were pretreated with sialidase.

The data are presented as the mean ± SD. A paired t test was used. *P<0.05, **P<0.01, ***P<0.001, and ****P<0.0001.

## **Fig. S6: Targeting sialoglycans during MDSC-like cell generation significantly reduces the expression of functional markers at the RNA level**

Suppressive MDSC-like cells were generated *in vitro* by coculture with A549 or A549-sia cancer cell lines as described in Figure 5. On Day 7, the CD33^+^ cells were sorted and processed for bulk RNA sequencing **(A-B)** or single-cell RNA (scRNA) sequencing **(C-H)**. **(A)** Volcano plot of genes differentially expressed in suppressive CD33^+^ cells generated from A549 or A549-Sia cancer cell lines and analyzed by bulk RNA sequencing. *n=4 per group*. **(B)** Gene Ontology (GO) enrichment analysis of the top 10 up- and downregulated gene sets found in suppressive CD33^+^ cells generated from A549-sia cells compared to those from the parental A549 cell line from **(A)**. **(C)** Heatmap showing the top differentially expressed (marked) genes in the scRNA-seq dataset for each cluster. The gene expression intensity is shown as a color gradient from purple (low) to yellow (high). **(D)** Seurat analysis of the scRNAseq dataset projected in UMAP subdivided into individual donor and treatment groups. MDSC-like cells were grouped into A549-generated MDSC-like cells (green, upper panel) and A549-sia MDSC-like cells (pink, lower panel). **(E)** A549 (green) and A549-Sia (pink) cells generated MDSC-like cells in each cluster. **(F)** Violin plots showing the relative MDSC signature scores of MDSCs from Alshetaiwi ^35^ subclustered with A549- and A549-sia-generated MDSC-like cells. The numbers in brackets indicate the number of overlying genes found in our scRNA dataset. **(G)** Gene Ontology (GO) enrichment analysis of the top 5 upregulated gene sets found in each cluster as a dot plot. The color coding indicates the adjusted p values, and the dot size is proportional to the mean normalized enrichment score (NES) found in the listed pathway.

The data are presented as the mean. The Wilcoxon rank sum test (F) was used. *P<0.05, **P<0.01, ***P<0.001, and ****P<0.0001

## **Fig. S7: CCL2 expression promotes the suppressive myeloid cell phenotype in humans and mice**

**(A)** Cytokines found in the supernatant of murine MDSC:T-cell cocultures at the endpoint of the experiment are shown in Fig. 3. Z scores were calculated for each cytokine. MDSCs were left untreated and pretreated with sialidase or Siglec-E*. n=3 donors per group*. **(B)** MCP-1/CCL2 found in the supernatant of human primary CD33^+^:CD8^+^ cell cocultures at the endpoint of the experiment from Fig. 4H. CD33^+^ cells were untreated or pretreated with sialidase. Z scores were calculated for each cytokine and are shown on a color scale from blue to red. *n=3 donors per group*. **(C)** Graphical summary: MDSCs express Siglec receptors and are highly sialylated, leading to activation (e.g., expression of CCL2). Suppressive MDSCs lead to decreased T-cell infiltration and promote tumor growth. Blocking Siglec receptors and reducing sialoglycans decreases MDSC suppressive function. As a result, T cells can infiltrate the TME and slow tumor growth.

The data are presented as the mean ± SD. One-way ANOVA was used. *P<0.05, **P<0.01, ***P<0.001, and ****P<0.0001.
