# Supplementary material for: Engagement of sialylated glycans with Siglec receptors on suppressive myeloid cells inhibits anticancer immunity via CCL2
Source: Cell Mol Immunol. 2024 Mar 6;21(5):495–509. doi: 10.1038/s41423-024-01142-0 (PMC11061307; doi:10.1038/s41423-024-01142-0)
Supplement: Supplementary file 9 — Supplementary Table [file 41423_2024_1142_MOESM9_ESM.docx]

## **Table S1: Flow cytometry antibodies**

| Target | Clone | Fluorochrome | Manufacturer | Catalog Number |
| --- | --- | --- | --- | --- |
| CD11b | ICRF44 | eFluor710 | eBioscience | 46-0118-42 |
| CD11b | M1/70 | APC-Cy7 | BioLegend | 101226 |
| CD11b | M1/70 | AF647 | BD Biosciences | 557686 |
| CD11c | N418 | BV421 | BioLegend | 117330 |
| CD11c | N418 | FITC | BioLegend | 117306 |
| CD14 | M5E2 | BV650 | BioLegend | 301836 |
| CD15 | HI98 | AF488 | BioLegend | 301910 |
| CD16 | B73.1 | APC-Cy7 | BioLegend | 360709 |
| CD19 | SJ25C1 | eFluor780 | eBioscience | 47-0198-42 |
| CD19 | 6D5 | BV570 | BioLegend | 115535 |
| CD19 | 1D3 | BB515 | BD Biosciences | 564509 |
| CD20 | 2H7 | eFluor780 | eBioscience | 47-0209-42 |
| CD25 | PC61.5 | PE-Cy5.5 | eBioscience | 35-0251-82 |
| CD3 | SK7 | eFluor780 | eBioscience | 47-0036-42 |
| CD3 | HIT3A | FITC | BioLegend | 300306 |
| CD3 | 145-2C11 | BUV805 | BD Biosciences | 741895 |
| CD3 | 145-2C11 | PE | BioLegend | 100308 |
| CD33 | WM53 | BV786 | BD Biosciences | 740974 |
| CD33 | AC104.3E3 | PE | Miltenyi Biotec | 130-113-349 |
| CD4 | SK3 | PE | eBioscience | 12-0047-42 |
| CD4 | GK1.5 | BUV496 | BD Biosciences | 612952 |
| CD4 | RM4-5 | BV605 | BioLegend | 100548 |
| CD45 | HI30 | BV510 | BioLegend | 304036 |
| CD45 | 2D1 | PerCP-Cy5.5 | Invitrogen | 45-9459-42 |
| CD45 | 30-F11 | BUV395 | BD Biosciences | 564279 |
| CD56 | CMSSB | eFluor780 | eBioscience | 47-0567-42 |
| CD8 | SK1 | APC | BioLegend | 344722 |
| CD8 | 53-6.7 | eFluor 450 | eBioscience | 48-0081-82 |
| CD8a | 53-6.7 | PE-Cy7 | eBioscience | 25-0081-82 |
| F4/80 | BM8 | AF647 | BioLegend | 123122 |
| GzmB | NGZB | PE-eFluor610 | eBioscience | 61-8898-82 |
| HLA DR | L243 | eFluor450 | eBioscience | 9048-9952-120 |
| Ki67 | SolA15 | AF532 | eBioscience | 58-5698-82 |
| Ly-6C | HK1.4 | PerCP | BioLegend | 128028 |
| Ly-6C | HK1.4 | PE | BioLegend | 128008 |
| Ly-6G | 1A8 | BUV563 | BD Biosciences | 612921 |
| Ly-6G | 1A8 | BV421 | BioLegend | 127627 |
| MHCII | M5/114.15.2 | BV510 | BioLegend | 107636 |
| NKp46 | 29A14 | BUV661 | BD Biosciences | 741678 |
| pSTAT3 | 13A3-1 | PE | BioLegend | 651004 |
| Siglec-10 | 5G6 | PE | BioLegend | 347604 |
| Siglec-5 | 1A5 | APC | BioLegend | 352006 |
| Siglec-7 | 6-434 | PE | BioLegend | 339204 |
| Siglec-9 | K8 | AF647 | BioLegend | 351510 |
| Siglec-E | M1304A01 | PE | BioLegend | 677104 |
| Siglec-F | E50-2440 | PE | BD Biosciences | 552126 |
| Siglec-G | SH2.1 | APC | Invitrogen | 17-5833-82 |
| Siglec-H | 551 | PE | BioLegend | 129605 |

## **Table S2: Patient characteristics**

| Gender | Age | Tumor type | Stage | Medication | Used for |
| --- | --- | --- | --- | --- | --- |
| female | 47 | NSCLC | IIB | none | Suppression assay |
| male | 49 | Lung squamous cell carcinoma | IB | none | Suppression assay |
| male | 51 | Lung adenocarcinoma | IIA | none | Siglec staining |
| male | 55 | Lung adenocarcinoma | IIIA | none | Lectin staining |
| male | 63 | Lung adenocarcinoma | IA2 | none | Lectin staining |
| female | 67 | Lung adenocarcinoma | IIB | none | Lectin staining |
| female | 68 | Lung adenocarcinoma | IIIB | none | Siglec staining |
| male | 70 | Lung adenocarcinoma | IA | none | Lectin staining |
| male | 70 | Lung squamous cell carcinoma | IA3 | none | Siglec staining |
| male | 70 | Lung adenocarcinoma | IB | none | Suppression assay |
| male | 71 | Lung adenocarcinoma | IIA | none | Siglec staining |
| female | 73 | Lung adenocarcinoma | IIIB | none | Lectin staining |
| male | 73 | Lung adenocarcinoma | IIA | none | Siglec staining |
| female | 73 | Lung adenocarcinoma | IIIB | neoadjuvant | Suppression assay |
| female | 73 | Lung adenocarcinoma | IB | none | Suppression assay |
| male | 74 | Lung adenocarcinoma | IIB | none | Lectin staining |
| male | 76 | NSCLC | IV | none | Siglec staining |
| male | 76 | Lung adenocarcinoma | IB | none | Suppression assay |
| male | 78 | Lung squamous cell carcinoma | IA3 | none | Siglec staining |
| female | 79 | Lung adenocarcinoma | IA | none | Lectin staining |
| female | 79 | Lung adenocarcinoma | IIB | none | Lectin staining |
| male | 79 | Lung adenocarcinoma | IA3 | none | Siglec staining |
| male | 79 | Lung adenocarcinoma | IIB | none | Siglec staining |
| female | 83 | Lung adenocarcinoma | IA2 | none | Siglec staining |
